# Peritoneal metastasis in pancreatic cancer: molecular mechanisms, microenvironmental remodeling, and emerging intraperitoneal interventions

**DOI:** 10.3389/fmolb.2026.1854674

**Published:** 2026-06-17

**Authors:** Junnan Chen, Kexin Li, Yuming Zou, Jinzu Yang, Lianjun Xing, Kunmin Xiao

**Affiliations:** 1 Department of Oncology, Longhua Hospital, Shanghai University of Traditional Chinese Medicine, Shanghai, China; 2 Department of Gastroenterology, Longhua Hospital, Shanghai University of Traditional Chinese Medicine, Shanghai, China; 3 Department of Traditional Chinese Medicine, Peking Union Medical College Hospital, Chinese Academy of Medical Sciences & Peking Union Medical College, Beijing, China; 4 Department of General Internal Medicine, Longhua Hospital, Shanghai University of Traditional Chinese Medicine, Shanghai, China

**Keywords:** pancreatic cancer, peritoneal metastasis, molecular mechanisms, microenvironment, intraperitoneal therapy

## Abstract

Pancreatic cancer remains one of the most lethal malignancies, and its frequent spread to the peritoneum significantly compromises patient survival and quality of life. Peritoneal metastasis arises from a complex interaction between tumor intrinsic features and the unique anatomical and immune environment of the peritoneal cavity. This review systematically elucidates the stepwise process of peritoneal dissemination, including initial cell detachment, epithelial-to-mesenchymal transition, resistance to anoikis, immune evasion, adhesion to the mesothelium, and angiogenesis. We then highlight four interconnected regulatory hubs that drive these events: metabolic reprogramming that supports tumor survival and reinforces immune suppression; extracellular vesicles that enable intercellular communication and premetastatic niche formation; cancer stem cells that confer phenotypic flexibility and therapy resistance; and the dynamically remodeled immune microenvironment that facilitates tumor colonization. Finally, we critically assess emerging locoregional treatment strategies, namely normothermic, hyperthermic, and pressurized intraperitoneal chemotherapy, designed to overcome the peritoneal-plasma barrier and improve clinical outcomes. Furthermore, we evaluate the potential synergistic effects of integrating these strategies with immunotherapy. By synthesizing current knowledge of the molecular and cellular mechanisms underlying peritoneal metastasis in pancreatic cancer, this review offers a conceptual framework for developing precision-targeted interventions against this devastating condition.

## 1 Introduction

Pancreatic cancer (PC) is one of the most severe malignant tumors globally, with a 5-year survival rate of approximately 10% and an extremely poor prognosis (Avula et al., 2020a). The latest data indicate that in 2021, there were approximately 508,532 new cases worldwide, leading to approximately 505,752 deaths. By 2044, the annual number of new cases and deaths worldwide is expected to exceed 875,000, underscoring the impending substantial public health burden (Li et al., 2024).

Anatomically, PC can be classified into pancreatic head cancer and pancreatic body or tail cancer. Histologically, pancreatic ductal adenocarcinoma (PDAC) is the most common subtype. PC is characterized by rapid progression and an insidious onset; consequently, approximately 80% of patients are diagnosed at an advanced stage when curative surgery is no longer feasible. Although radical resection remains the only potentially curative treatment, only about 20% of patients are eligible for surgical intervention. Compounding this challenge, PC exhibits aggressive behavior and high metastatic potential. PC frequently disseminates via lymphatic and hematogenous spread, perineural invasion, direct extension into adjacent organs, and peritoneal seeding, all of which severely compromise patient survival and quality of life (Stoop et al., 2025). The peritoneum is the second most common site of metastasis in patients with PC, surpassed only by the liver (Shukla et al., 2025). It typically results from the seeding of detached pancreatic cancer cells (PCCs) in the peritoneal cavity and is frequently accompanied by ascites formation. Peritoneal metastasis (PM) exhibits a molecular signature distinct from that of hematogenous or lymphatic dissemination, and its presence is associated with significant morbidity, including bowel obstruction, ascites, and urinary obstruction (Wu et al., 2025).

Given the distinct molecular signature and substantial clinical burden of PM, this review provides a systematic summary of the molecular and immunological mechanisms underlying PM, with particular emphasis on the stepwise cascade, core regulatory networks, and remodeling of the peritoneal immune microenvironment. Additionally, this review discusses the persistent clinical challenges associated with PM and examines the rationale for locoregional intraperitoneal therapies and their potential integration with immunotherapy. In summary, this review establishes a theoretical foundation and outlines future research directions for precision therapy targeting PM in PC.

## 2 Anatomical and biological basis of peritoneal metastasis

**FIGURE 1:**
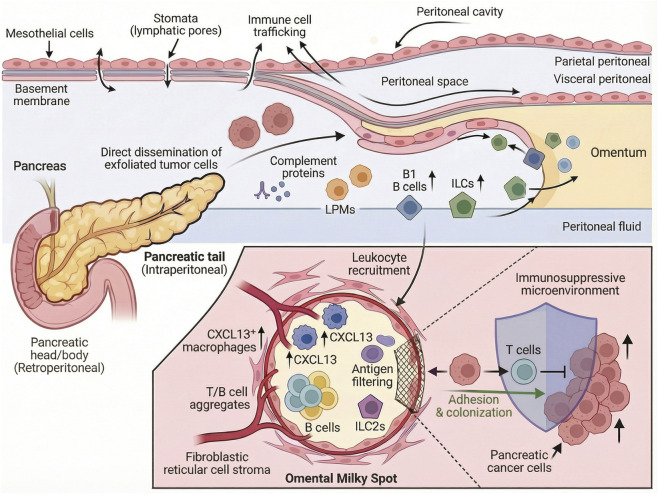
Structural and cellular components of the peritoneal microenvironment. Peritoneal mesothelial cells feature stomata that facilitate the migration of immune cells. The pancreatic tail is intraperitoneal, whereas the head and body are retroperitoneal, a distinction that influences the spread of the pancreatic cancer cells. Peritoneal fluid contains LPMs and ILCs. Omental milky spots, characterized by CXCL13^+^ macrophages, T/B Cells, and fibroblastic stroma, establish an immunosuppressive niche for tumor adhesion and colonization. Abbreviations: LPMs, large peritoneal macrophages; ILCs, innate lymphoid cells; CXCL, C-X-C motif chemokine ligand.

The development of PM in PC is governed not only by tumor-intrinsic factors but also by the distinctive anatomical and immunological landscape of the peritoneal cavity (Figure 1).

Anatomically, the peritoneum comprises a monolayer of mesothelial cells overlying a basement membrane, forming both parietal and visceral layers that enclose the potential peritoneal space. This serous membrane is interspersed with specialized stomata, small pores between mesothelial cells that facilitate lymphatic drainage and immune cell trafficking, particularly in the omentum and diaphragm. The pancreas exhibits a unique anatomical configuration: while the tail lies intraperitoneally, the head and body reside in the retroperitoneal space. This anatomical dichotomy may promote divergent metastatic pathways for exfoliated PCCs, rendering intraperitoneal regions more susceptible to direct dissemination into the peritoneal fluid (Gaballah et al., 2024).

Physiologically, early studies established that the peritoneal cavity contains a small amount of sterile fluid that distributes leukocytes throughout the abdominal cavity, serving dual roles as a lubricant and a dynamic immune reservoir (Sedlacek et al., 2013). Subsequent work has further revealed that this fluid contains complement proteins and a distinct leukocyte repertoire, including large peritoneal macrophages, B1 B Cells, and innate lymphoid cells (ILCs). These cells are sustained by stromal niches in the omentum, a visceral adipose tissue historically described as the “policeman of the abdomen” for its ability to filter peritoneal fluid and support local immune responses (Liu et al., 2021). The omentum houses milky spots, specialized lymphoid clusters resembling secondary lymphoid organs but devoid of follicular dendritic cell networks. These structures are highly vascularized and composed of C-X-C motif chemokine ligand 13 (CXCL13)-producing macrophages, T and B Cell aggregates, and ILC2s, all embedded in a fibroblastic reticular cell stroma. The unique organization of milky spots creates immune gateways that filter peritoneal antigens and recruit leukocytes (Okabe, 2021). These features collectively foster an immunosuppressive niche that facilitates PCCs adhesion, immune evasion, and colonization.

## 3 The multistep cascade of peritoneal metastasis

**FIGURE 2:**
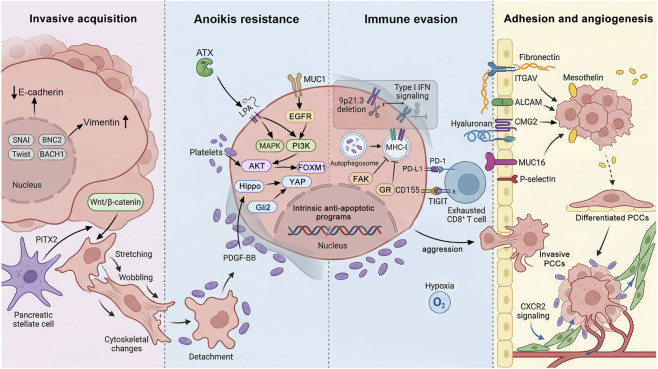
Key molecules involved in the four-step peritoneal metastasis cascade of pancreatic cancer. From left to right, the four panels correspond to step 1 (invasive acquisition), step 2 (anoikis resistance), step 3 (immune evasion), and step 4 (adhesion and angiogenesis). The cascade progresses from EMT-driven detachment through mechanisms of anoikis resistance and immune evasion, culminating inmesothelial adhesion, invasion, and angiogenesis, which sustain metastatic outgrowth. Key molecules mediating each transition are illustrated in the figure. Abbreviations: ALCAM, activated leukocyte cell adhesion molecule; ATX, autotaxin; AKT, protein kinase B; BACH1, BTB and CNC homology 1; BNC2, basonuclin 2; CMG2, capillary morphogenesis gene 2; CXCR2, C-X-C motif chemokine receptor 2; EGFR, epidermal growth factor receptor; FOXM1, forkhead box M1; FAK, focal adhesion kinase; Gli2, glioma-associated oncogene family zinc finger 2; GR, glucocorticoid receptor; Hippo-YAP, Hippo-Yes-associated protein pathway; ITGAV, integrin alpha V; LPA, lysophosphatidic acid; MAPK, mitogen-activated protein kinase; MUC, mucin; MHC-I, major histocompatibility complex class I; PDGF-BB, platelet-derived growth factor BB; PD-L1, programmed death-ligand 1; PI3K, phosphoinositide 3-kinase; PITX2, pituitary homeobox 2; PCC, pancreatic cancer cell; SNAI, snail family transcriptional repressor; TIGIT, T Cell immunoreceptor with Ig and ITIM domains; Twist, twist-related protein; Wnt/β-catenin, Wingless-related integration site/β-catenin.

PM in PC involves a complex, multistep process comprising four critical stages: (1) PCCs initially detach from the primary tumor, acquiring a migratory phenotype that facilitates their dissemination into the peritoneal cavity. (2) These disseminated cells must subsequently survive in suspension, developing resistance to detachment-induced apoptosis to persist as free-floating entities. (3) Surviving cells evade host immune surveillance and actively suppress anti-tumor immunity to prevent clearance within the peritoneal environment. (4) Upon reaching the peritoneal surface, they adhere to and disrupt the mesothelial barrier, invade the underlying matrix, and promote angiogenesis to secure vascularization for metastatic outgrowth. The representative molecules involved in each of these four steps are summarized in Figure 2.

### 3.1 Detachment and invasive acquisition

The initial stage involves PCC detachment from the primary tumor and acquisition of an invasive phenotype, primarily driven by epithelial-mesenchymal transition (EMT). During EMT, PCCs lose epithelial characteristics and gain mesenchymal traits, marked by E-cadherin downregulation and vimentin upregulation, thereby enhancing their migratory and invasive capabilities (Kamakura et al., 2022). Core EMT-inducing transcription factors (EMT-TFs), notably the Snail family transcriptional repressors (SNAI) and Twist-related proteins (Twist), orchestrate this process. *In vitro* and xenograft studies have demonstrated that silencing these factors significantly impairs PCC migration, invasion, and stemness (Kaşıkcı et al., 2020).

Beyond these well-established EMT-TFs, additional transcription factors contribute to EMT regulation during PC progression. The basic leucine zipper transcription factor BTB and CNC homology 1 (BACH1) promotes EMT and metastasis by repressing epithelial genes and activating mesenchymal inducers. Its high expression in human tumor specimens correlates with poor prognosis, although a causal relationship remains unconfirmed (Sato et al., 2020). The paired-like homeodomain transcription factor Pituitary homeobox 2 (PITX2) is highly expressed in pancreatic stellate cells (PSCs), where it activates Wnt/β-catenin signaling to promote EMT and tumor progression. Elevated stromal PITX2 expression in patient samples is associated with poor clinical outcomes (Wu et al., 2023). Initial evidence also suggests that the zinc finger transcription factor Basonuclin 2 (BNC2) may promote invasion by upregulating collagen type III alpha 1 chain, though this finding requires independent validation before BNC2 can be considered a *bona fide* driver of PM (Li et al., 2025).

In summary, detachment and the adoption of an invasive phenotype center on EMT, orchestrated by core transcription factors including SNAI and Twist, alongside additional regulators such as BACH1, PITX2, and BNC2. Most of these factors have been functionally characterized *in vitro* or in xenograft models, with limited validation in human PM specimens, and their potential as therapeutic targets remains to be established. Subsequent sections explore the regulatory mechanisms driving these EMT-related changes, including metabolic reprogramming, extracellular vesicle networks, and cancer stem cell plasticity.

### 3.2 Anoikis resistance

Upon detaching from the primary tumor, PCCs must overcome anoikis, a specialized form of programmed cell death triggered by the loss of extracellular matrix attachment. Acquiring anoikis resistance enables PCCs to survive in the peritoneal cavity, thereby representing the second essential step in PM.

Multiple mechanisms contribute to this adaptation. Platelets interact directly with PCCs to activate protein kinase B (AKT) signaling, upregulating forkhead box M1 (FOXM1); in patient samples, FOXM1 expression is higher in metastatic lesions than in primary tumors and correlates with poor prognosis (Ernesti-Soldatkin et al., 2026). *In vitro*, platelet-derived growth factor BB suppresses anoikis under anchorage-independent conditions by activating the Hippo pathway and its downstream effector yes-associated protein (YAP) (Li et al., 2021). Malignant ascites also constitutes a pro-survival microenvironment. In a PDAC mouse model, autotaxin (ATX) is highly abundant in ascites and promotes PCC migration and survival via lysophosphatidic acid-mediated activation of mitogen-activated protein kinase (MAPK) and phosphoinositide 3-kinase (PI3K)/AKT signaling. Furthermore, pharmacological inhibition of ATX suppresses peritoneal dissemination and reduces ascites accumulation (Jinno et al., 2021). Beyond microenvironmental signals, PCCs use intrinsic survival strategies. Cell line experiments identified a ceRNA network (MIR4435-2HG/miR-513a-5p) that upregulates the anti-apoptotic gene BCL2L1, and clinical cohort analyses associated elevated expression of this axis with poor prognosis (Zhu et al., 2024). Knockout of glioma-associated oncogene family zinc finger 2 (Gli2) in PCC lines induces anoikis by increasing YAP1 phosphorylation and reducing its nuclear translocation; in a murine tail-vein injection model, Gli2-knockout cells formed fewer metastases with increased apoptosis (Yu et al., 2022). *In vitro* studies further demonstrate that mucin 1 (MUC1) promotes anoikis resistance through both ligand-dependent and ligand-independent mechanisms by activating epidermal growth factor receptor (EGFR)/PI3K signaling under anchorage-independent conditions; this mechanism is further supported by xenograft models (Bose et al., 2023). Computational analyses of human tumor cohorts (TCGA and GEO) have identified numerous anoikis-related genes associated with adverse outcomes. At the protein level, EGFR and matrix metalloproteinase expression were significantly correlated with survival in patient samples, while common driver mutations such as Kirsten rat sarcoma viral oncogene homolog (KRAS), tumor protein p53 (TP53), and cyclin-dependent kinase inhibitor 2A (CDKN2A) were computationally linked to enhanced anoikis resistance (Zhang et al., 2023).

In summary, anoikis resistance enables PCCs to survive in suspension through platelet-derived signals, ascites-derived factors, intrinsic anti-apoptotic pathways, and genetic alterations. The majority of these mechanisms have been elucidated in cell lines, murine models, and retrospective bioinformatic analyses, yet lack substantial prospective validation in human PM specimens. Thus, anoikis resistance represents a rate-limiting step in the metastatic cascade whose disruption could halt peritoneal dissemination, but the translational gap between preclinical targets and clinically validated therapies remains substantial. This capacity is further sustained by metabolic adaptations and the inherent resilience of cancer stem cells (CSCs), as examined in subsequent sections.

### 3.3 Immune evasion

Beyond acquiring anoikis resistance to survive in the peritoneal cavity, disseminated PCCs must simultaneously overcome host immune defenses to successfully establish colonization. This process involves multiple mechanisms through which PCCs actively modulate the microenvironment to suppress anti-tumor immunity.

PCCs harbor intrinsic genetic and molecular alterations that disrupt immune recognition. In genetically engineered mouse models, co-deletion of the type I interferon (IFN) gene cluster and cyclin-dependent kinase inhibitor 2A/B at the 9p21.3 locus impairs type I IFN signaling, thereby reducing CD8^+^ T cell-mediated surveillance and promoting both metastasis and immunotherapy resistance. Subsequent analysis of TCGA and COMPASS trial datasets validated the clinical relevance of these deletions in PC (Barriga et al., 2022). Beyond such genetic lesions, PCCs downregulate surface expression of major histocompatibility complex class I (MHC-I) via selective autophagy targeting MHC-I for lysosomal degradation. This mechanism was demonstrated in murine syngeneic transplantation models and is supported by correlative evidence from resected PDAC patient specimens, which show intracellular MHC-I localization (Yamamoto et al., 2020). Focal adhesion kinase (FAK) further suppresses MHC-I expression through a nuclear-dependent mechanism. In KPC mouse models and patient-derived cell lines, FAK inhibits immunoproteasome activity and antigen presentation, and its deletion restores MHC-I expression and enhances T-cell recognition; proteomic analyses of human PDAC cell lines confirm the cross-species conservation of this regulatory network (Canel et al., 2023). Glucocorticoid receptor (GR) signaling represents another mechanism of immune evasion by transcriptionally repressing MHC-I while upregulating programmed death-ligand 1 (PD-L1). In a tissue microarray analysis of 101 PC patients, GR expression correlated with elevated PD-L1, reduced MHC-I, reduced CD8^+^ T Cell infiltration, and poor prognosis; furthermore, plasma cortisol levels were elevated in patients compared with healthy controls. However, the therapeutic efficacy of GR inhibitors combined with immune checkpoint blockade has not been evaluated in clinical trials (Deng et al., 2021). Beyond these MHC-I-targeted strategies, PCCs also induce T-cell exhaustion. In KPC mouse models and human PDAC genomic datasets, oncogenic KRAS drives CD155 overexpression, particularly in the context of combined KRAS and TP53 mutations. The engagement of CD155 with TIGIT (T Cell immunoreceptor with immunoglobulin and immunoreceptor tyrosine-based inhibition motif domains) on tumor-infiltrating CD8^+^ T Cells promotes T-cell exhaustion. Consistent with this immunomodulatory role, KRAS ablation in orthotopic PDAC models triggers increased infiltration of CD4^+^ and CD8^+^ T Cells and elevated MHC gene expression, indicating that oncogenic KRAS actively suppresses antitumor immunity. In human PDAC, tumors with lower KRAS transcriptional activity exhibit greater T-cell infiltration and higher expression of immune checkpoint molecules, reinforcing the clinical relevance of this mechanism. Preclinical studies combining TIGIT blockade with PD-1 inhibition and CD40 agonism demonstrated enhanced antitumor immunity, although data on therapeutic responses in humans are not yet available (Freed-Pastor et al., 2021; Ischenko et al., 2021).

In summary, immune evasion in PC during the metastatic cascade is mediated by genetic disruption of IFN signaling, KRAS-driven suppression of antitumor immunity, downregulation of MHC-I, and upregulation of inhibitory checkpoints such as PD-L1 and CD155. These mechanisms operate at the level of individual disseminated tumor cells and enable them to escape immune detection during their transit through the peritoneal fluid. The subsequent colonization of the peritoneal surface requires a further layer of immune subversion that is spatially orchestrated within the unique peritoneal immune microenvironment, where resident stromal and immune components are actively remodeled to support metastatic outgrowth. Most of the mechanisms discussed here have been characterized in genetically engineered mouse models, syngeneic transplantation systems, and patient-derived cell lines. Clinical correlative evidence, including tissue microarray analyses and serum biomarker measurements, underscores their clinical relevance. However, therapeutic interventions targeting these pathways have not yet been validated in prospective clinical trials for PM. Thus, while the molecular circuitry of immune evasion during dissemination is being increasingly elucidated at the preclinical level, its translational potential remains contingent upon rigorous clinical testing. The coordinated execution of these immune evasion strategies is closely coupled to the broader regulatory networks that govern PM, as explored in the subsequent sections.

### 3.4 Cell adhesion molecules and angiogenesis

Following immune evasion and survival in the peritoneal cavity, disseminated PCCs must establish stable footholds by anchoring to the mesothelial lining, disrupting this protective barrier, penetrating the underlying extracellular matrix, and subsequently promoting angiogenesis to secure vascular support for nascent metastatic foci.

PCCs achieve initial anchoring through specific interactions between surface adhesion molecules and ligands on peritoneal mesothelial cells or exposed extracellular matrix components. Integrin alpha V (ITGAV, also known as CD51) serves as a critical adhesion molecule that anchors PCCs to the peritoneal lining; its knockdown significantly reduces adhesion to fibronectin and impairs peritoneal carcinomatosis formation in murine models. An analysis of a tissue microarray comprising samples from 209 PDAC patients revealed that high ITGAV expression was associated with shorter survival, supporting its clinical relevance (Kemper et al., 2021). Activated leukocyte cell adhesion molecule (ALCAM) plays a dual role in this process, acting as a receptor on both PCCs and mesothelial cells to mediate heterotypic adhesion via homophilic interactions. In cohorts of patients with gastric cancer and PC, ALCAM transcript levels were significantly elevated in those whose tumors subsequently progressed to PM, and high ALCAM expression was associated with shorter PM-free survival (Yang et al., 2023). Capillary morphogenesis gene 2 (CMG2) is upregulated in PC tissues relative to normal pancreatic tissue, as demonstrated by immunohistochemical analysis of a 153-patient cohort and TCGA datasets. *In vitro*, CMG2 promotes PM by facilitating cell-matrix adhesion through binding to hyaluronan and activating the EGFR/FAK pathways (Fang et al., 2024). MUC16 contributes to peritoneal spread by enhancing PCC binding to endothelial cells and to P-selectin expressed on the peritoneal mesothelium; in human datasets, MUC16 expression correlates with metastasis, although direct interventional evidence remains limited (Lakshmanan et al., 2022).

Following initial adhesion, PCCs actively disrupt the intact mesothelial layer. Studies using *in vitro* peritoneal models highlight mechanistic heterogeneity in this process: highly invasive PCCs such as MIA PaCa-2 migrate across the mesothelium, disrupt the mesothelial sheet, and invade the underlying matrix, whereas moderately differentiated cells like BxPC3 proliferate on the mesothelial surface and form sheets that replace the original mesothelium without vertical invasion (Odagiri et al., 2021). The ability of PCCs to form aggregates also modulates colonization efficiency. In studies employing cell lines and nude mouse models, mesothelin (MSLN) interacts with MUC16 to promote PCC aggregation; conversely, excessive secretion of soluble MSLN inhibits this process and diminishes peritoneal colonization (Ewa et al., 2024). MSLN also promotes endothelial cell recruitment and activation at the colonization stage, enhancing tumor vascular density in murine peritoneal transplantation models; notably, this activity depends on the Y318 residue and appears to be independent of MUC16 binding, as well as nuclear factor κB (NF-κB) and MAPK pathway activation (Avula et al., 2020b). Host chemokine signaling further augments angiogenesis. In murine models, C-X-C motif chemokine receptor 2 (CXCR2), expressed on endothelial cells and innate immune cells, drives this process in response to tumor-derived ligands, and deletion of CXCR2 results in reduced microvessel density within pancreatic tumors (Purohit et al., 2021).

In summary, mesothelial adhesion, invasion, and angiogenesis form a sequential cascade in which PCCs utilize adhesion molecules such as ITGAV, ALCAM, CMG2, and MUC16 to anchor to the peritoneal lining, disrupt the mesothelial barrier through diverse invasive strategies, and subsequently promote angiogenesis to secure vascular support. Among these molecules, the roles of ALCAM and CMG2 are substantiated by both experimental data and evidence from patient tissues, while the contributions of MSLN and CXCR2 to PM have been investigated predominantly in animal models. Thus, while the molecular determinants of peritoneal anchoring and vascularization are increasingly elucidated, their validation as therapeutic targets in patients with PM remains incomplete. The broader regulatory networks that drive these adhesive and invasive phenotypes are explored in the subsequent sections.

## 4 Core regulatory hubs driving peritoneal metastasis

**FIGURE 3:**
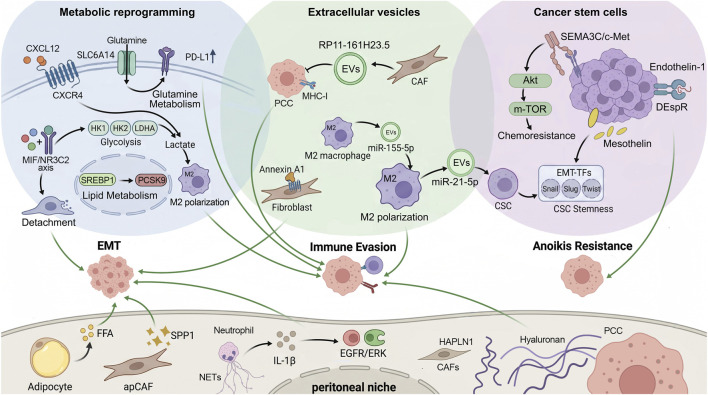
Integrated regulatory network of peritoneal metastasis. The four central hubs, namely, metabolic reprogramming, extracellular vesicles, cancer stem cells, and the peritoneal niche, converge to drive three outcomes, specifically EMT and invasion, anoikis resistance, and immune evasion, thereby enabling peritoneal colonization. Abbreviations: Akt, protein kinase B; CAF, cancer-associated fibroblast; c-Met, cellular-mesenchymal epithelial transition factor; CSC, cancer stem cell; CXCL12, C-X-C motif chemokine ligand 12; CXCR4, C-X-C motif chemokine receptor 4; DEspR, dual endothelin-1/signal peptide receptor; EGFR, epidermal growth factor receptor; EMT-TFs, epithelial-mesenchymal transition transcription factors; ERK, extracellular signal-regulated kinase; EVs, extracellular vesicles; FFA, free fatty acid; HAPLN1, hyaluronan and proteoglycan link protein 1; HK1, hexokinase 1; HK2, hexokinase 2; LDHA, lactate dehydrogenase A; MHC-I, major histocompatibility complex class I; MIF, macrophage migration inhibitory factor; m-TOR, mechanistic target of rapamycin; NETs, neutrophil extracellular traps; NR3C2, nuclear receptor subfamily 3 group C member 2; PCC, pancreatic cancer cell; PCSK9, proprotein convertase subtilisin/kexin type 9; PD-L1, programmed death-ligand 1; SEMA3C, semaphorin 3C; SLC6A14, solute carrier family 6 member 14; Slug, snail family transcriptional repressor 2; Snail, snail family transcriptional repressor 1; SPP1, secreted phosphoprotein 1; SREBP1, sterol regulatory element-binding protein 1; Twist, twist-related protein.

While the preceding chapter delineated the sequential steps of PM, the present section explores four interconnected regulatory hubs that collectively drive the metastatic cascade: metabolic reprogramming, extracellular vesicles (EVs), CSCs, and the remodeled immune microenvironment. These hubs do not operate in isolation; rather, they form a coordinated network that integrates EMT, anoikis resistance, and immune evasion to facilitate peritoneal colonization, as summarized in Figure 3. The subsequent subsections elucidate each hub in detail, with accompanying figures illustrating the molecular components and pathways corresponding to each.

### 4.1 Metabolic reprogramming

**FIGURE 4:**
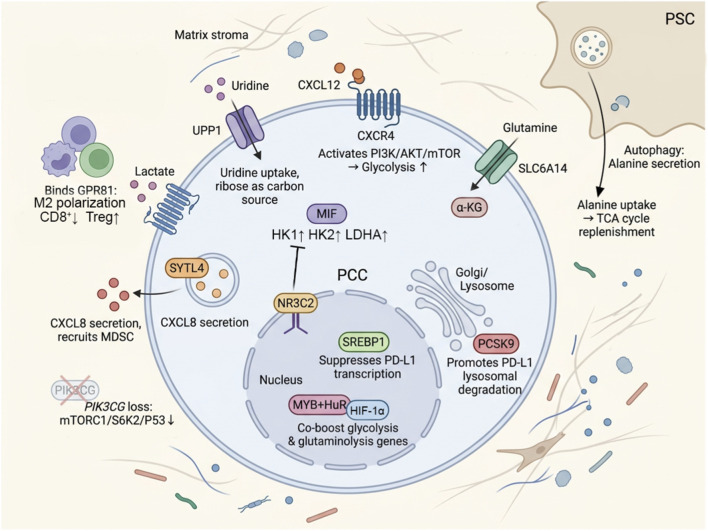
Interconnected layers of metabolic reprogramming driving peritoneal metastatic colonization. The schematic illustrates intrinsic metabolic flexibility (spanning glucose, glutamine, and lipid pathways), direct coupling of metabolism to immune checkpoint regulation, and stromal ecosystem remodeling. Abbreviations: α-KG, α-ketoglutarate; AKT, protein kinase B; CXCL, C-X-C motif chemokine ligand; CXCR, C-X-C motif chemokine receptor; GPR81, G protein-coupled receptor 81; HK, Hexokinase; HIF-1α, hypoxia-inducible factor 1-alpha; HuR, human antigen R; LDHA, lactate dehydrogenase A; MDSC, myeloid-derived suppressor cell; MIF, macrophage migration inhibitory factor; mTOR, mechanistic target of rapamycin kinase; mTORC1, mTOR complex 1; M2, alternatively activated macrophage phenotype; MYB, MYB proto-oncogene; NR3C2, nuclear receptor subfamily 3 group C member 2; PCC, pancreatic cancer cell; PCSK9, proprotein convertase subtilisin/kexin type 9; PD-L1, programmed death-ligand 1; P53, tumor protein p53; PI3K, phosphoinositide 3-kinase; PSC, pancreatic stellate cell; S6K2, ribosomal protein S6 kinase beta 2; SLC6A14, solute carrier family 6 member 14; SREBP1, sterol regulatory element-binding protein 1; SYTL4, synaptotagmin-like protein 4; TCA, tricarboxylic acid cycle; UPP1, uridine phosphorylase 1.

To adapt to the hypoxic, nutrient-deprived, and desmoplastic microenvironment of the peritoneal cavity, PCCs undergo extensive metabolic reprogramming that drives tumor growth, potentiates metastatic potential, and establishes an immunosuppressive landscape. This metabolic rewiring can be characterized by three interrelated dimensions: intrinsic metabolic flexibility, metabolic checkpoint coupling, and stromal immune metabolic crosstalk, as illustrated in Figure 4.

Upon entering the nutrient-deprived peritoneal cavity, PCCs leverage intrinsic metabolic flexibility to sustain energy production. During glucose deprivation, PCCs upregulate uridine phosphorylase 1 (UPP1) to utilize ribose from extracellular uridine as an alternative carbon source. In patient samples, UPP1 expression is significantly higher in tumor tissue than in non-tumor tissue and correlates with poor prognosis. Furthermore, uridine has been detected in tumor interstitial fluid, confirming its availability within the tumor microenvironment (Nwosu et al., 2023). Stromal PSCs reinforce this metabolic dependency by secreting CXCL12, which binds to CXCR4 on PCCs and activates PI3K/AKT/mammalian target of rapamycin (mTOR) signaling to enhance glycolysis (Lu et al., 2022). This glycolytic program is further modulated by the macrophage migration inhibitory factor (MIF)/nuclear receptor subfamily 3 group C member 2 (NR3C2) axis. In patient samples, MIF expression positively correlates with glycolytic gene expression and metabolite levels, whereas NR3C2 expression exhibits an inverse correlation. In cell line and xenograft models, MIF activates MAPK-ERK signaling to upregulate key glycolytic enzymes such as hexokinase 1, hexokinase 2, and lactate dehydrogenase A (LDHA); additionally, this axis modulates lipid metabolic gene expression, positioning it as a central hub that coordinates multiple metabolic pathways (Yang et al., 2024). In parallel, sterol regulatory element-binding protein 1 (SREBP1), the master regulator of lipogenesis, modulates PD-L1 expression through two distinct mechanisms: direct transcriptional repression and upregulation of proprotein convertase subtilisin/kexin type 9 (PCSK9), which promotes lysosomal PD-L1 degradation. In patient samples, serum lipid levels correlate with anti-PD-1 therapy response, and SREBP1 expression is inversely associated with PD-L1 in tumor tissues (Lao et al., 2025). Together, the MIF/NR3C2 axis and the SREBP1-PCSK9 pathway illustrate how metabolic rewiring integrates glucose utilization, lipid homeostasis, and immune checkpoint regulation to support tumor progression.

In addition to the metabolic adaptations involving glucose and lipids, glutamine metabolism constitutes another critical axis for sustaining PCC survival and immune evasion. The solute carrier family 6 member 14 (SLC6A14) mediates glutamine uptake, thereby generating α-ketoglutarate to activate mTOR/NF-κB signaling. This activation transcriptionally upregulates PD-L1 and promotes synaptotagmin-like protein 4-dependent CXCL8 secretion, recruiting immunosuppressive myeloid cells. In patient cohorts, high SLC6A14 expression is associated with poor prognosis (Kang et al., 2025). The proto-oncogene MYB, stabilized by the RNA-binding protein human antigen R under hypoxia, cooperates with hypoxia-inducible factor 1-alpha (HIF-1α) to enhance glycolytic and glutaminolytic gene expression. In orthotopic xenograft models, MYB knockout significantly reduced tumorigenicity and metabolic activity, and HIF-1α overexpression failed to rescue this phenotype (Anand et al., 2023). Notably, phosphatidylinositol-4,5-bisphosphate 3-kinase catalytic subunit gamma (PIK3CG) deficiency suppresses the mTORC1/S6K2/P53 axis, reducing glutaminase 2 (GLS2) expression and disrupting glutamine catabolism; paradoxically, this disruption suppresses pyroptosis, thereby facilitating tumor progression. In patient tissues, PIK3CG and GLS2 are downregulated, and low PIK3CG expression correlates with poor prognosis (Han et al., 2026).

Beyond sustaining PCC survival, these metabolic pathways actively reshape the immune landscape through multiple mechanisms. Beyond enhancing glycolysis, the CXCR4/CXCL12 axis directly contributes to immune evasion. In KPC mouse models, CXCL12 secreted by activated PSCs was shown to reduce cytotoxic CD8^+^ T lymphocyte infiltration and drive M2 polarization of tumor-associated macrophages; furthermore, intraperitoneal delivery of CXCR4-antagonizing nanoparticles enhanced T-cell infiltration and reduced metastasis (Xie et al., 2020). This dual function of CXCL12, enhancing glycolysis while simultaneously promoting an immunosuppressive microenvironment, exemplifies the close coupling between metabolic reprogramming and immune evasion. The end product of glycolysis, lactate, further reinforces this immunosuppressive milieu by binding the G-protein coupled receptor 81 on immune cells. This interaction polarizes tumor-associated macrophages toward the immunosuppressive M2 phenotype characterized by the upregulation of arginase 1 (ARG1), interleukin-10, and vascular endothelial growth factor, while concurrently suppressing CD8^+^ T Cell function and promoting regulatory T Cell differentiation. In patient tissues, elevated global lysine lactylation, a post-translational modification driven by lactate, correlates with reduced CD8^+^ T Cell infiltration, increased macrophage accumulation, and poor immunotherapy outcomes (Sun et al., 2025; Gao et al., 2025). Lactate production is driven by LDHA, the same glycolytic enzyme upregulated by the MIF/NR3C2 axis. Beyond producing lactate, LDHA also generates the oncometabolite L-2-hydroxyglutarate, which promotes cancer stem cell properties and directly inhibits T-cell proliferation and migration. Elevated L-2-hydroxyglutarate has been detected in serum from PC patients, though the core mechanistic findings derive from preclinical systems (Gupta et al., 2021). This lactate-driven immunosuppression is amplified by SLC6A14-mediated glutamine metabolism, which coordinates tumor bioenergetics with immune evasion (Kang et al., 2025). Furthermore, PSCs undergo autophagy to secrete alanine, which PCCs utilize to fuel the tricarboxylic acid cycle, sustaining proliferation and immunosuppressive factor production under nutrient stress (Amrutkar and Gladhaug, 2021).

In summary, intrinsic metabolic flexibility spanning glucose, glutamine, and lipid pathways enables disseminated PCCs to survive nutrient stress, evade immune surveillance, and establish a permissive metastatic niche within the peritoneal cavity. Several metabolic targets discussed here, including UPP1, SLC6A14, SREBP1, and the MIF/NR3C2 axis, have been validated in patient samples at the expression level and via correlative analyses, supporting their clinical relevance. The integration of CXCL12-mediated PSC-PCC crosstalk with lactate-driven M2 polarization illustrates how metabolic reprogramming is inextricably linked to immune suppression in the peritoneal cavity. However, the therapeutic tractability of these targets in PM remains largely untested. The highly adaptive nature of tumor metabolism, characterized by redundant fuel sources and compensatory pathways, suggests that single-agent metabolic inhibition is unlikely to achieve durable responses. Rational combination strategies integrating metabolic inhibitors with chemotherapy, immunotherapy, or locoregional delivery approaches represent a promising therapeutic avenue, yet they await prospective clinical testing in patients with PM from PC.

### 4.2 Extracellular vesicles

**FIGURE 5:**
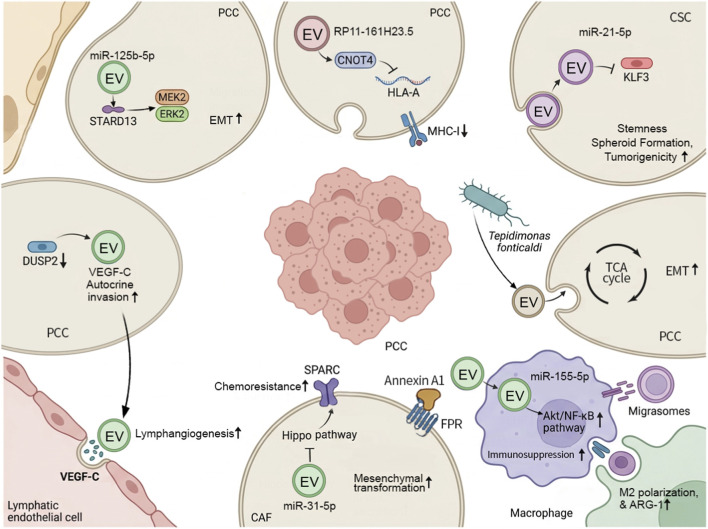
Extracellular vesicle-mediated crosstalk in peritoneal metastasis. Extracellular vesicles from PCCs, CAFs, M2 macrophages, and microbes transport miRNAs, lncRNAs, and proteins to remodel the peritoneal niche, driving EMT, immune evasion, chemoresistance, and angiogenesis. Abbreviations: Akt, protein kinase B; ARG1, arginase 1; CAF, cancer-associated fibroblast; CNOT4, CCR4-NOT transcription complex subunit 4; DUSP2, dual-specificity phosphatase 2; EMT, epithelial-mesenchymal transition; EV, extracellular vesicle; FPR, formyl peptide receptor; HLA-A, major histocompatibility complex, class I, A; KLF3, Kruppel-like factor 3; M2, alternatively activated macrophage phenotype; MEK2, mitogen-activated protein kinase 2; MHC-I, major histocompatibility complex class I; NF-κB, nuclear factor κB; PCC, pancreatic cancer cell; SPARC, secreted protein acidic and cysteine rich; STARD13, StAR-related lipid transfer domain containing 13; TCA, tricarboxylic acid cycle; VEGF-C, vascular endothelial growth factor C.

Tumor-derived EVs are pivotal for priming distant metastatic sites. PDAC-derived exosomes are taken up by resident macrophages in organs such as the liver, where they deliver MIF and induce fibronectin deposition, facilitating myeloid cell recruitment and the establishment of a pre-metastatic niche (Inzunza and Del Valle, 2025). This organ-specific signaling exemplifies how EV-mediated communication contributes to the site-specific tropism of PC metastasis, underscoring the broad relevance of the “seed and soil” paradigm. An analogous mechanism operates within the peritoneal cavity, a growing body of evidence indicates that EVs derived from diverse cellular sources, such as PCCs, cancer-associated fibroblasts (CAFs), immune cells, and even microbes, orchestrate multiple stages of PM through a complex intercellular communication network. Figure 5 schematically illustrates these EV-mediated communication networks and their contributions to peritoneal metastatic progression.

PCC-derived EVs play a central role in disseminating the metastatic phenotype. In isogenic PCC lines derived from a single primary tumor, exosomes secreted by highly invasive PC-1.0 cells significantly enhanced migration, invasion, and EMT in their weakly invasive PC-1 counterparts following internalization. These exosomes are enriched in microRNA-125b-5p, which directly targets the mRNA of the tumor suppressor StAR-related lipid transfer domain containing 13 and activates the MEK2/ERK2 signaling pathway, thereby transferring pro-metastatic traits between PCC subpopulations (Wu et al., 2020). Beyond such direct communication among PCCs, tumor-derived EVs also shape the immune landscape. In co-culture systems and murine xenograft models, PCC-derived EVs transfer miR-155–5p to macrophages, where it targets ETS homologous factor (EHF) and activates the Akt/NF-κB pathway, promoting polarization into the immunosuppressive M2 phenotype. Analysis of 55 patient tissue samples revealed an inverse correlation between miR-155–5p and EHF expression, supporting the clinical relevance of this axis (Wang and Gao, 2021). Expanding the repertoire of EV-related structures, migrasomes represent a distinct class of vesicles generated by migrating PCCs. In murine models, these structures are taken up by macrophages, inducing M2 polarization and ARG1 expression, thereby establishing an immune-refractory microenvironment in the peritoneal cavity (Zhang et al., 2024).

Alongside PCC-derived EVs, CAFs represent another critical source of EVs that remodel the peritoneal niche. The communication between CAFs and PCCs is bidirectional. CAF-secreted EVs encapsulate the lncRNA RP11-161H23.5, and upon uptake by PCCs, this lncRNA recruits CCR4-NOT transcription complex subunit 4 to promote HLA-A mRNA degradation, thereby downregulating MHC-I surface expression and enabling evasion of CD8^+^ T cell-mediated cytotoxicity. In CKP mouse models and humanized PDX models, engineered EVs loaded with siRNA targeting RP11-161H23.5 restored HLA-A expression and sensitized tumors to dual immune checkpoint blockade (Yao et al., 2024). Conversely, PCC-derived EVs transfer miR-31–5p to CAFs, inhibiting the Hippo pathway and promoting the secretion of secreted protein acidic and cysteine rich (SPARC), which in turn activates ERK signaling in PCCs to reinforce chemoresistance and survival. In patient tissue microarrays, high miR-31–5p expression was associated with shorter overall survival (OS) (Qin et al., 2024). The protein Annexin A1 further exemplifies this crosstalk. Experiments using the MIA PaCa-2 cell line demonstrated that Annexin A1-enriched EVs from PCCs activate formyl peptide receptors on fibroblasts and endothelial cells, triggering mesenchymal transition and enhancing cell motility, thereby facilitating tumor-stroma communication (Novizio et al., 2020).

In parallel, immune cell-derived EVs amplify these pro-tumorigenic signals. In nude mouse xenograft models, M2 macrophage-derived EVs encapsulate miR-21–5p, which targets Kruppel-like factor 3 in PC stem cells to enhance stemness, sphere formation, and tumorigenic potential (Chang et al., 2022). Meanwhile, in KPC and KDC mouse models, suppression of dual-specificity phosphatase 2 (DUSP2) in PCCs leads to increased secretion of EV-associated vascular endothelial growth factor C (VEGF-C), which acts on lymphatic endothelial cells to promote lymphangiogenesis while also exerting autocrine effects on PCCs to enhance invasiveness. In 54 patient tissues, DUSP2 expression was found to be downregulated in premalignant and malignant lesions and inversely correlated with VEGF-C expression (Wang et al., 2020). Moreover, microbial components also contribute to this EV-mediated network. In 15 PDAC patient tissue samples, the composition of the EV-associated microbiome differed between tumor and matched normal tissues, with specific microbiota such as Tepidimonas enriched in tumors. The supernatant of Tepidimonas fonticaldi, which contains bacteria-derived EVs, was shown to promote PCC proliferation, migration, and EMT and to alter tricarboxylic acid cycle-related metabolite profiles *in vitro* (Jeong et al., 2020).

In summary, EVs derived from multiple cellular sources, including PCCs of varying metastatic potential, CAFs, M2 macrophages, and even microbial communities, harbor a heterogeneous repertoire of pro-tumorigenic cargoes, such as miRNAs, lncRNAs, and proteins (Romano et al., 2021). Through this integrated network of intercellular communication, EVs synergistically promote EMT, immune evasion, chemoresistance, and angiogenesis, orchestrating the stepwise progression of PM. Several EV cargoes discussed here, including miR-155–5p, miR-31–5p, and DUSP2/VEGF-C, correlate with clinical outcomes in patients, underscoring their translational significance. However, the clinical application of EVs as biomarkers or therapeutic targets in PM remains in an early stage of development. Furthermore, the functional redundancy within this network suggests that targeting individual EV cargoes may be insufficient, whereas strategies aimed at EV biogenesis or uptake may offer greater therapeutic efficacy. Addressing these translational barriers will be essential for unlocking the diagnostic and therapeutic potential of EVs in patients with PM from PC.

### 4.3 Cancer stem cells

**FIGURE 6:**
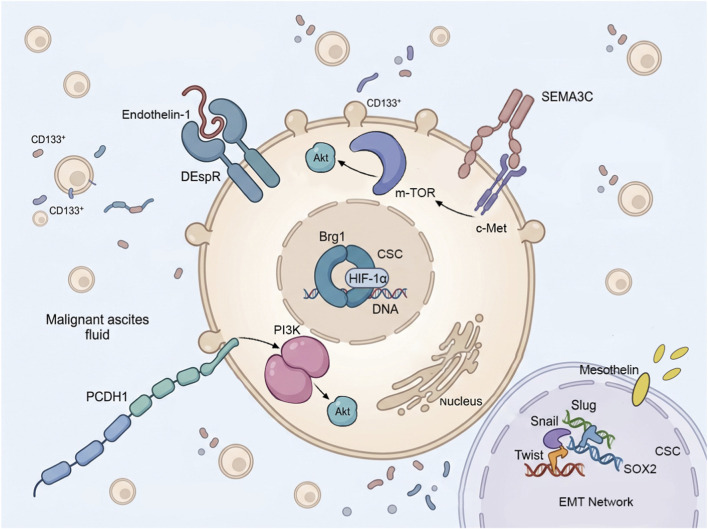
Cancer stem cell-driven mechanisms in peritoneal metastasis. CSCs promote peritoneal dissemination through anoikis resistance, hypoxic adaptation, chemoresistance, and EMT-coupled plasticity. Abbreviations: Akt, protein kinase B; Brg1, Brahma-related gene 1; c-Met, cellular-mesenchymal epithelial transition factor; CSC, cancer stem cell; DEspR, dual endothelin-1/signal peptide receptor; EMT, epithelial-mesenchymal transition; HIF-1α, hypoxia-inducible factor 1-alpha; m-TOR, mechanistic target of rapamycin kinase; PCDH1, protocadherin-1; PI3K, phosphoinositide 3-kinase; SEMA3C, semaphorin 3C; SOX2, SRY-box transcription factor 2; Snail, snail family transcriptional repressor 1; Slug, snail family transcriptional repressor 2; Twist, twist-related protein.

CSCs constitute a functionally distinct subpopulation within pancreatic tumors, characterized by their capacity for self-renewal, tumor initiation, and therapeutic resistance. As central drivers of PM, CSCs orchestrate multiple key steps of the metastatic cascade, including survival under detached conditions, adaptation to hostile microenvironments, and acquisition of invasive capabilities. Importantly, CSCs are present in malignant ascites and actively participate in peritoneal dissemination. CD133, a well-established marker of pancreatic CSCs, has been detected at high levels in ascites-derived exosomes from patients with advanced PC. In a cohort of 19 patients, the presence of highly glycosylated CD133 in ascites-derived exosomes correlated with prolonged OS. Immunohistochemistry confirmed CD133 expression on the membrane and in the cytoplasm of PCCs in both primary and peritoneal metastatic lesions, confirming their tumor cell origin (Sakaue et al., 2019). Moreover, CSCs exhibit considerable phenotypic diversity. In PCC lines and murine metastasis models, epithelial-like CSCs, as exemplified by Panc89 Holoclone cells, are characterized by high SRY-box transcription factor 2 expression, rapid proliferation, and robust self-renewal capacity; notably, these cells tend to form relatively few but large tumors with a preferential tropism for the peritoneum (Philipp et al., 2024). The key molecular pathways and functional attributes of CSCs that collectively drive PM are outlined in Figure 6.

A hallmark of CSCs in PM is their ability to resist anoikis, a form of apoptosis triggered by detachment from the extracellular matrix. In a single xenograft study, subpopulations of PCCs that survive anoikis were enriched in CSC phenotypes and exhibited elevated expression of the dual endothelin-1/signal peptide receptor (DEspR). Functional targeting of DEspR with a humanized antibody effectively impeded peritoneal spread and prolonged median OS in this model. In human PC tissue microarrays, DEspR was detected in stage II-IV primary and metastatic tumors, providing a rationale for clinical translation, though independent replication remains pending (Gromisch et al., 2021). Beyond resistance to detachment-induced cell death, CSCs also adapt to the hypoxic microenvironment encountered during dissemination. In spontaneous PDAC mouse models and cell line experiments, the chromatin remodeler Brahma-related gene 1 (Brg1) sustains CSC properties by facilitating HIF-1α binding to hypoxia-responsive elements of target genes. Brg1 deletion significantly suppressed PDAC growth, metastasis, and stemness. In human PDAC samples, low BRG1 expression correlated with downregulation of hypoxia, cell cycle, and stemness-related gene sets (Araki et al., 2023). Moreover, CSCs acquire intrinsic chemoresistance through distinct signaling cascades. In orthotopic and PM mouse models, semaphorin 3C (SEMA3C) was demonstrated to promote CSC maintenance and confer resistance to gemcitabine via activation of the c-Met/Akt/mTOR pathway; furthermore, its knockdown synergized with gemcitabine plus nab-paclitaxel to suppress peritoneal tumor burden. In patient samples, high SEMA3C expression was associated with tumor invasion, PM, and poor prognosis (Tomizawa et al., 2023). Similarly, an initial report has suggested that protocadherin-1 (PCDH1), a stemness-associated oncogene overexpressed in PDAC, may support CSC self-renewal by activating the PI3K-AKT signaling axis. In patient tissues, PCDH1 expression was verified by qRT-PCR and immunohistochemistry, though functional validation derives from cell line and animal model experiments (Yang et al., 2025). Collectively, these findings demonstrate that CSCs use a multifaceted survival strategy integrating anoikis resistance, hypoxic adaptation, and chemoresistance to persist and proliferate within the hostile environment of the peritoneal cavity.

The properties of CSCs are intricately linked to the EMT program, forming a bidirectional regulatory network that underpins cellular plasticity. In PCC lines, key EMT transcription factors, including Snail, Slug, and Twist, were shown not only to drive the acquisition of mesenchymal traits but also to induce and maintain stemness. Silencing these factors downregulated stem cell markers such as CD24, CD44, CD133, and CXCR4, thereby compromising tumor sphere formation (Kaşıkcı et al., 2020). Likewise, in cell line and xenograft models, MSLN, which functions as an EMT inducer, was shown to concurrently enrich CSC populations. Its knockout diminished clonogenicity and tumorigenicity, whereas its overexpression enhanced these stemness-associated properties and increased the IC50 for gemcitabine (Hu et al., 2024). This mutual reinforcement between EMT and stemness enables PCCs to dynamically transition between phenotypic states, thereby facilitating both dissemination from the primary tumor and subsequent colonization of distant sites.

In summary, these findings establish CSCs as pivotal nodes that orchestrate adaptive survival mechanisms, chemoresistance, and phenotypic plasticity to drive PM in PC. The identification of key molecular pathways governing CSC functions, including DEspR-mediated anoikis resistance, Brg1/HIF-1α-dependent hypoxic adaptation, SEMA3C-driven chemoresistance, PCDH1-mediated PI3K-AKT signaling, and EMT-regulatory networks, reveals multiple vulnerabilities that represent promising therapeutic targets. Among these, CD133 and SEMA3C are supported by patient-derived correlative evidence, whereas DEspR and PCDH1 have been characterized primarily in individual preclinical studies and await independent validation. Brg1 and MSLN are supported by both functional studies and patient sample analyses, though prospective clinical data remain unavailable. Thus, while the CSC-driven mechanisms underlying peritoneal dissemination are increasingly elucidated at the preclinical level, the translational development of CSC-targeted therapies for patients with PM remains in its early stages and requires rigorous clinical evaluation.

### 4.4 Intraperitoneal immune microenvironment

**FIGURE 7:**
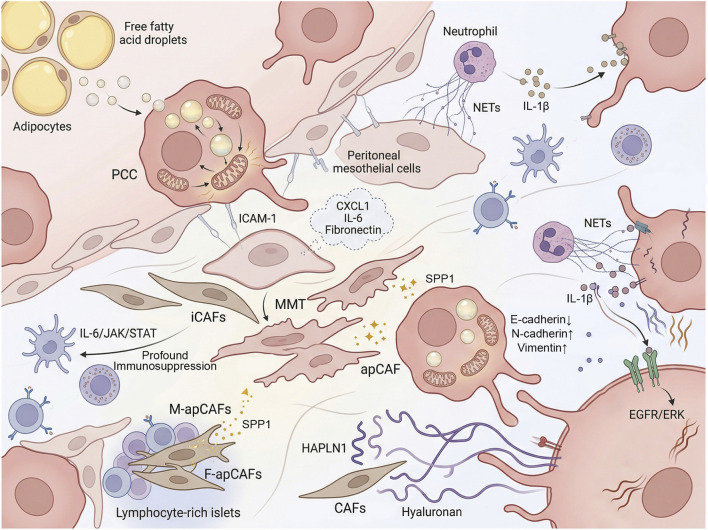
Remodeling of the peritoneal immune microenvironment during metastatic colonization. PCCs orchestrate a supportive niche through metabolic crosstalk with adipocytes, MMT-derived CAFs, and immune dysregulation. Key processes include free fatty acid transfer from adipocytes, mesothelial senescence and CAF transdifferentiation, CAF subset heterogeneity (iCAFs and apCAFs), NET-mediated EMT, and impaired adaptive immunity. Abbreviations: apCAFs, antigen-presenting cancer-associated fibroblasts; CXCL, C-X-C motif chemokine ligand; EGFR, epidermal growth factor receptor; ERK, extracellular signal-regulated kinase; F-apCAFs, lymphocyte-rich region antigen-presenting cancer-associated fibroblasts; HAPLN1, hyaluronan and proteoglycan link protein 1; iCAFs, inflammatory cancer-associated fibroblasts; ICAM-1, intercellular adhesion molecule 1; IL, interleukin; JAK, Janus kinase; MMT, mesothelial-mesenchymal transition; M-apCAFs, mesothelial-like antigen-presenting cancer-associated fibroblasts; NETs, neutrophil extracellular traps; PCC, pancreatic cancer cell; SPP1, secreted phosphoprotein 1; STAT, signal transducer and activator of transcription.

The intraperitoneal immune microenvironment represents a complex ecosystem formed by PCCs and local stromal and immune cells, whose establishment is a dynamic, multi-step process. Within this niche, PCCs actively remodel the peritoneal “soil” by modulating the functional states of and driving the phenotypic transformation of mesothelial cells, fibroblasts, and immune cells, thereby establishing a metastatic milieu that supports tumor dissemination, immune evasion, and therapeutic resistance. The cellular heterogeneity and functional crosstalk within the peritoneal immune microenvironment are illustrated in Figure 7.

Peritoneal adipose tissue, particularly within the omentum, has been implicated in the PM of various malignancies, as the abundant adipocytes within milky spots provide a metabolically rich and structurally supportive niche for tumor dissemination (Kim et al., 2026). Although PC is not explicitly highlighted in these observations, direct evidence from co-culture systems and murine models demonstrates that adipocytes actively interact with PCCs through metabolic crosstalk, whereby adipocytes supply free fatty acids that are internalized by PCCs, sequestered as lipid droplets, and subsequently oxidized to fuel invasive and metastatic activities. Immunohistochemical observations of human PC tissues have revealed alterations in adipocyte size adjacent to tumor cells, though the core mechanistic findings derive from cell line experiments (Cai et al., 2023). This metabolic reprogramming, together with the phenotypic alterations adipocytes undergo in response to tumor-derived signals, suggests that peritoneal adipocytes contribute to the PM of PC by actively remodeling the local microenvironment. Beyond this metabolic interplay, peritoneal mesothelial cells, as the first-line barrier of the peritoneum, also undergo profound phenotypic changes. Under physiological conditions, these cells exert anti-tumor effects by inducing apoptosis in PCCs through soluble factors such as intercellular adhesion molecule 1. However, upon exposure to PCCs, they can undergo senescence and acquire a senescence-associated secretory phenotype characterized by the secretion of CXCL1, IL-6, and fibronectin, a profile that paradoxically promotes tumor adhesion and invasion. Critically, these cells can undergo mesothelial-mesenchymal transition and transdifferentiate into CAFs, thereby actively remodeling the extracellular matrix, enhancing tumor invasion, and creating a permissive environment for transcoelomic spread (Li and Guo, 2022).

These CAFs, derived in part from mesothelial transdifferentiation, represent a major component of the heterogeneous fibroblast population within the peritoneal microenvironment. The desmoplastic stroma in PC is not a static barrier but a dynamic structure composed of distinct cellular populations and spatial domains, wherein CAFs exhibit remarkable phenotypic and functional heterogeneity. Single-cell spatial analyses have identified distinct CAF subsets that form specialized niches. Inflammatory CAFs represent the most abundant CAF subset in the peritoneal microenvironment and may contribute to immune regulation via the interleukin-6/Janus kinase/signal transducer and activator of transcription (IL-6/JAK/STAT) pathway (Ramos et al., 2024). Antigen-presenting CAFs (apCAFs) comprise two spatially segregated subpopulations: one associated with mesothelial-like cells near PCCs (M-apCAFs), and another located in lymphocyte-rich regions (F-apCAFs). Notably, the mesothelial origin of M-apCAFs directly corroborates the active remodeling of the peritoneal “soil” by PCCs. In murine PM models, single-cell sequencing and spatial transcriptomic analyses revealed that both apCAF subsets upregulate secreted phosphoprotein 1 (SPP1), a factor that promotes primary tumor formation, PM, and therapeutic resistance. These findings were further validated in human PC and PM specimens by single-cell sequencing, spatial imaging, and immunohistochemistry (Chen et al., 2025). Furthermore, these fibroblasts secrete hyaluronan and proteoglycan link protein 1 (HAPLN1), which upregulates the expression of tumor necrosis factor receptor in the tumor microenvironment, thereby promoting hyaluronic acid production. In peritoneal carcinomatosis mouse models and PDAC cell lines, HAPLN1 was shown to drive EMT, stemness, and invasive capacity in PCCs while also facilitating metastasis by inducing M2 macrophage polarization and suppressing the production of pro-inflammatory cytokines. In patient cohorts, high HAPLN1 expression was associated with the basal subtype, enrichment of EMT-related gene sets, and shorter OS (Wiedmann et al., 2023).

Within the immunosuppressive stroma remodeled by these heterogeneous CAF subsets, the peritoneal immune landscape undergoes profound dysregulation characterized by both the absence of effective anti-tumor immunity and the presence of pro-tumor inflammatory cells. In syngeneic mouse models carrying Kras G12D and Trp53 R172H mutations, aggressive PDAC subtypes prone to peritoneal dissemination commonly exhibit deficiencies in dendritic cells and T Cells, contributing to profound immunosuppression that allows PCCs to colonize the peritoneal cavity and proliferate without effective immune intervention (Tanji et al., 2026). In addition to the deficit in adaptive immunity, the innate immune compartment, particularly neutrophils, plays an active role in promoting metastatic progression. In a multicenter retrospective study of patients, ascitic samples from patients with PC and PM showed significantly higher neutrophil proportions and counts compared with ascitic samples from cases associated with portal hypertension. This elevation is attributed to the inflammatory response and increased vascular permeability induced by direct tumor infiltration, serving as a distinguishing feature of PM-associated ascites relative to other etiologies (Yeo et al., 2025). These neutrophils can actively promote tumor progression through the formation of neutrophil extracellular traps. In co-culture experiments involving PCC lines and nude mouse subcutaneous xenograft models, these traps were demonstrated to release IL-1β, which activates the EGFR/ERK pathway in PCCs, resulting in E-cadherin downregulation and mesenchymal marker upregulation, thereby driving PCC migration, invasion, and EMT progression. Immunohistochemical analysis of tissue samples from 150 PDAC patients revealed that the abundance of these traps correlated positively with phosphorylated EGFR levels and negatively with E-cadherin expression, underscoring the clinical relevance of this mechanism (Jin et al., 2021).

In summary, the peritoneal metastatic microenvironment in PC is an ecosystem actively sculpted by PCCs through dynamic interactions with stromal and immune components. This process begins with the remodeling of the mesothelial barrier, accompanied by metabolic crosstalk with peritoneal adipocytes that supply free fatty acids to fuel tumor progression. The resulting CAF population exhibits heterogeneity, with distinct subsets contributing to immune regulation, matrix remodeling, and tumor promotion. Within this remodeled stroma, the immune landscape becomes profoundly dysregulated, characterized by deficiencies in adaptive immunity alongside neutrophil-mediated tumor promotion through trap formation. Among the molecular and cellular components discussed here, HAPLN1, apCAF subsets, and neutrophil extracellular traps are supported by both functional studies and patient-derived correlative evidence, whereas the mechanistic understanding of adipocyte-PCC metabolic crosstalk is derived primarily from co-culture systems and awaits clinical validation. Currently, single-cell sequencing data specific to PM of PC remain limited, and in-depth dissection of this network will provide crucial insights for developing more effective targeted therapies. The identification of clinically actionable vulnerabilities within this complex ecosystem represents a key priority for future research.

## 5 Clinical challenges in peritoneal metastasis

The clinical management of PM in PC is hindered by late detection, the limited diagnostic utility of cross-sectional imaging, a lack of validated biomarkers, and the pharmacokinetic limitations of systemic chemotherapy. These clinical challenges underscore the urgent need for improved diagnostic strategies, validated biomarkers, and evidence-based patient selection algorithms specific to peritoneal disease.

### 5.1 Detection of occult peritoneal metastasis

Conventional contrast-enhanced computed tomography (CT) remains the standard imaging modality for staging PC, yet it consistently fails to detect small-volume peritoneal disease. Studies have demonstrated that standard CT protocols have insufficient sensitivity for identifying minimal peritoneal deposits, with up to 10%–30% of patients deemed resectable by imaging actually harboring occult PM at the time of surgical exploration (Stefanidis et al., 2006). Despite significant advances in imaging technology, the diagnosis of PC and its peritoneal spread remains delayed in the vast majority of patients, contributing to the disease’s dismal prognosis (Zha et al., 2018).

Given the limitations of CT, staging laparoscopy (SL) has long been advocated as a complementary tool. SL identified occult intra-abdominal metastases in 28% of patients with potentially resectable tumors and 33% of patients with locally advanced disease, and it was significantly more likely than open exploration to identify these occult deposits (32% vs. 10%, p = 0.018) (Contreras et al., 2009). More recent studies have also shown that the absolute risk reduction for non-therapeutic laparotomy in patients undergoing SL is 12.5% (Jambor et al., 2022). It is to be noted that SL cannot detect vascular invasion, lymph node involvement and deep hepatic metastases, and hence must be used in addition to other imaging modalities (De et al., 2016). A number of modern imaging advances aim to improve detection. For example, dual-energy CT with material density (MD) image reconstruction has shown promise in enhancing early detection of peritoneal carcinomatosis, with one study reporting that experienced readers achieved 98% sensitivity on 65 keV + MD images versus 90% on conventional 65 keV images alone (p = 0.02) (Pisuchpen et al., 2024). Yet even these emerging technologies have not fully resolved the problem of detecting low-volume, miliary peritoneal disease.

### 5.2 Diagnostic biomarkers

Carbohydrate antigen 19–9 (CA19-9) is the most commonly used serum biomarker for PC, but it has major limitations in detecting and monitoring PM. It shows 70%–80% sensitivity and 80%–90% specificity, with false negatives in lewis blood group-negative individuals and false positives in benign conditions such as cholangitis, pancreatitis, and biliary obstruction (Mal et al., 2023).

Currently, Circulating tumor DNA (ctDNA) is a promising blood-based biomarker for occult PM in PC. In a prospective study, ctDNA was detected in 41.0% of patients with occult metastases compared with 14.6% of those without (P = 0.001) and independently predicted occult metastases (OR = 3.113; P = 0.039). When combined with carcinoembryonic antigen and CA19-9, ctDNA achieved 66.7% sensitivity and 81.6% specificity (Hata et al., 2021). A novel approach focuses on the detection of tumor-derived DNA in peritoneal lavage specimens. Studies have shown that tumor-derived DNA is detectable even in patients with PC without overt or occult metastases. The presence of tumor-derived DNA in peritoneal lavage may serve as a molecular residual disease marker, aiding in the prediction of subclinical micrometastases and estimate the risk of recurrence and poor survival outcomes (Chiba et al., 2022). Peritoneal lavage cytology, performed as part of SL, has low sensitivity in detecting occult metastases, however, peritoneal lavage tumour DNA can be used as a more sensitive supplementary detection method for staged laparoscopy. A study suggests that peritoneal lavage tumor DNA could serve as an adjunct biomarker during staging laparoscopy for the more sensitive diagnosis of peritoneal dissemination (Suenaga et al., 2021).

### 5.3 Limitations of systemic chemotherapy

A fundamental limitation of systemic chemotherapy for PM stems from the unique pharmacokinetic properties of the peritoneal cavity. The peritoneal-plasma barrier, composed of the submesothelial stroma and the endothelial glycocalyx lining submesothelial capillaries, impedes the translocation of systemically administered agents from the systemic circulation into the peritoneal cavity (Ceelen et al., 2020). Although modern regimens have improved outcomes for a subset of patients, the prognosis for patients with PM remains poor, the median OS is still less than 1 year (Chakrabarti et al., 2022).

The Japanese clinical practice guideline for PDAC with peritoneal dissemination recommends systemic chemotherapy for eligible patients and suggests considering intraperitoneal chemotherapy for those without massive ascites (Satoi et al., 2022). In terms of specific first-line regimens, retrospective data have shown that gemcitabine plus nab-paclitaxel (GnP: gemcitabine plus albumin-bound paclitaxel) and modified FOLFIRINOX (mFFX: fluorouracil, leucovorin, irinotecan, and oxaliplatin) significantly improve outcomes compared with older regimens. In patients with PM, GnP or mFFX achieved an objective response rate of 28% versus 5%, a disease control rate of 73% versus 47%, a median progression-free survival of 6.1 versus 3.4 months, and a median OS of 10.1 versus 6.8 months (p < 0.001). The use of GnP or mFFX was identified as an independent factor associated with improved prognosis (HR = 0.32, p < 0.001) (Takeda et al., 2021). A large Canadian multicenter real-world cohort (Chord consortium) further confirmed the poor prognosis of PC patients with peritoneal spread. Among 1,161 patients with metastatic disease, PM was present in 14.6%, and these patients had a significantly shorter median OS compared to those without peritoneal involvement (3 vs. 7 months, p < 0.001). For those who received first-line chemotherapy, median OS was 7 months with FOLFIRINOX, 6 months with gemcitabine plus nab-paclitaxel, and 2 months with gemcitabine monotherapy, with combination regimens showing a significant survival advantage over gemcitabine alone (p = 0.002) (Pauls et al., 2020).

## 6 Emerging intraperitoneal therapeutic strategies

While the management of PM in PC remains challenging, intraperitoneal therapy offers potential clinical benefits for selected patients. Intraperitoneal chemotherapy overcomes the pharmacokinetic limitations imposed by the peritoneal-plasma barrier through the direct delivery of high concentrations of chemotherapeutic agents into the peritoneal cavity (Wu et al., 2025). The application of these therapies requires rigorous patient selection based on performance status, extent of peritoneal involvement, and response to prior systemic therapy; however, evidence supporting specific selection criteria for locoregional therapy remains limited, thereby necessitating individualized treatment plans formulated by a multidisciplinary team (Ganatra et al., 2026). The following sections summarize the clinical evidence supporting each approach, assess the heterogeneity of the data, and evaluate the integration of locoregional therapy and systemic immunotherapy.

### 6.1 Normothermic intraperitoneal chemotherapy

Normothermic intraperitoneal chemotherapy (NIPEC) is an increasingly adopted locoregional treatment strategy for PM in PC. By delivering chemotherapeutic agents directly into the peritoneal cavity, NIPEC achieves high local drug concentrations while minimizing systemic toxicity. When integrated with systemic chemotherapy, it significantly enhances drug penetration into peritoneal lesions and demonstrates promising clinical efficacy. Two principal combination strategies have been extensively investigated: intraperitoneal plus intravenous (i.p. + i. v.) regimens, and intraperitoneal plus combined intravenous and oral (i.p. + i. v. + p. o.) regimens. The key characteristics, efficacy outcomes, and safety profiles of representative clinical studies evaluating these strategies are summarized in Table 1.

**TABLE 1:** Normothermic intraperitoneal chemotherapy: High local drug exposure with minimized systemic toxicity.

Author/Year	Study Type	Regimen	Patient Population	Efficacy Outcomes	Safety/Conversion
Takahara et al. (2021)	Phase I	i.p. PTX + i.v. GnP	First-line; PS 0–1; peritoneal ± other mets	mPFS 5.4 months; RR 25%; Cytology- 67%	G3/4 neutropenia 58%; Port complications 33%
Yamada et al. (2020)	Phase I/II	i.p. PTX + i.v. GnP	First-line; PS 0–1; peritoneal only	mOS 14.5 months; 1 year OS 61%; RR 49%; Cytology- 39%	G3/4 neutropenia 70%; Conversion 17% (R0 88%)
Meguro et al. (2024)	Case report	i.p. PTX + i.v. GnP + CART	First-line; massive ascites; PS 2–3	OS 19 weeks, 36 weeks; Ascites resolved; Cytology conversion (1/2)	No prophylactic port; CART enabled chemo
Takahara et al. (2016)	Phase II	i.v.+i.p. PTX + S-1	GEM-refractory; malignant ascites; PS 0–2	mOS 4.8 months; Ascites resolved 69%; Cytology- 31%	G3/4 neutropenia 34%; Catheter infection 6%
Satoi et al. (2017a)	Phase II	i.v.+i.p. PTX + S-1	First-line; PS 0–1; peritoneal only	mOS 16.3 months; 1 year OS 62%; RR 36%; Cytology- 55%	G3/4 neutropenia 42%; Conversion 24% (R0 75%)
Satoi et al. (2017b)	Retrospective	i.v.+i.p. PTX + S-1 vs. conventional chemo	First-line; PS 0–1; peritoneal metastasis only	mOS 20 vs. 10 months (p = 0.004); Ascites at 1 year 25% vs. 62%	Conversion surgery 30% vs. 7% (p = 0.032); Grade 3/4 neutropenia 60% (study group)
Yamada et al. (2021)	Prospective	i.v.+i.p. PTX + S-1 or i.p. PTX + i.v. GnP	First-line; PS 0–1; peritoneal only	Conversion 20.3%; Surgical mOS 32.5 months; R0 81%	Time to surgery 9.0 months; Peritoneal recurrence 50%
Kitayama et al. (2016)	Case report	i.v.+i.p. PTX + S-1	Limited peritoneal mets; good PS	Pathologic fibrosis; R0 resection	Preop chemo 5 months; Recurrence 8 months post-surgery
Yamamoto et al. (2022)	Retrospective	i.p. PTX + i.v. GnP vs. conventional systemic chemo	First-line; PS 0–1; peritoneal only	mOS 17.9 vs. 10.2 months (p = 0.006); Cytology- 56%	Conversion 23% vs. 4%; Surgical mOS 27.4 months

Abbreviations: NIPEC, normothermic intraperitoneal chemotherapy; i. v., intravenous; i. p., intraperitoneal; PTX, paclitaxel; GnP, gemcitabine plus nab-paclitaxel; GEM, gemcitabine; S-1, oral fluoropyrimidine derivative; CART, cell-free and concentrated ascites reinfusion therapy; PS, performance status; mOS, median OS; mPFS, median progression-free survival; RR, response rate; R0, margin-negative resection; G3/4, grade 3 or 4; mo, months; wk, weeks.

The combination of intraperitoneal paclitaxel (i.p. PTX) with the GnP regimen has exhibited synergistic antitumor effects. A phase I study in patients with PC and PM reported a median progression-free survival of 5.4 months, an objective response rate of 25%, and a disease control rate of 75% (Takahara et al., 2021). A subsequent phase I/II trial involving 46 patients demonstrated a median OS of 14.5 months, with 1-year and 2-year survival rates of 61% and 32%, respectively. Notably, 18 of 46 patients (39%) achieved cytological conversion of peritoneal lavage, and malignant ascites completely resolved in 12 of 30 patients (40%) with baseline ascites (Yamada et al., 2020). Additionally, cell-free and concentrated ascites reinfusion therapy may serve as a valuable pre-treatment strategy to improve performance status, thereby enabling subsequent intraperitoneal chemotherapy in selected patients with massive malignant ascites (Meguro et al., 2024).

The regimen combining i. v./i.p. PTX with oral S-1 (a fluoropyrimidine derivative) has also shown substantial efficacy, particularly in gemcitabine-refractory patients with PM, yielding promising outcomes across different clinical settings. In patients with gemcitabine-refractory PC and malignant ascites, this regimen achieved a median OS of 4.8 months, with ascites reduction or resolution in 69% and cytological conversion in 31% of patients (Takahara et al., 2016). In contrast, a phase II trial in chemotherapy-naive patients with disease limited to PM reported even more robust results, including a median OS of 16.3 months and a 1-year survival rate of 62% (Satoi et al., 2017a). A single-institution retrospective study comparing the same regimen with conventional chemotherapy (gemcitabine or S-1 based) also demonstrated a median OS benefit (20 vs. 10 months, p = 0.004) and a higher conversion surgery rate (30% vs. 7%, p = 0.032) (Satoi et al., 2017b).

Conversion surgery was found to be feasible in selected patients following intraperitoneal paclitaxel-based therapy and was associated with improved postoperative survival. A pooled analysis of two prospective trials showed that among 79 patients, 16 (20.3%) underwent conversion surgery, with a median OS of 32.5 months from initial treatment and a median recurrence-free survival of 9.2 months after surgery (Yamada et al., 2021). A case report further demonstrated that even in a patient with limited PM and no radiographic primary tumor shrinkage, pathological complete response of peritoneal lesions was achieved, enabling R0 resection (Kitayama et al., 2016). A retrospective comparative study by Yamamoto and colleagues, which included 43 patients receiving i. p. PTX-containing regimens and 49 receiving standard systemic chemotherapy, revealed superior median OS (17.9 vs. 10.2 months, p = 0.006) and a higher conversion surgery rate (23% vs. 4%, p = 0.005) for the i. p. PTX group (Yamamoto et al., 2022).

The studies above highlight several patient selection criteria for intraperitoneal paclitaxel combined with systemic therapy. While most trials required a performance status of 0 or 1, the presence of massive ascites frequently precluded port placement, although ascites reinfusion made select patients eligible. Prior systemic therapy was also a significant prognostic factor; chemotherapy-naive patients demonstrated markedly prolonged survival compared to those who had already progressed on gemcitabine, with a median OS of 16.3 months versus 4.8 months. However, these data are derived from a heterogeneous mix of study designs, including phase I and II trials, retrospective analyses, and case reports. Inclusion criteria varied widely, with some studies excluding patients with any extrapulmonary metastases and others allowing those with liver involvement. Endpoints were similarly heterogeneous, ranging from dose-limiting toxicity to cytological conversion. Such heterogeneity complicates the direct comparison of results and highlights the need for standardized eligibility criteria in future studies.

### 6.2 Hyperthermic intraperitoneal chemotherapy

Hyperthermic intraperitoneal chemotherapy (HIPEC) combines regional drug delivery with hyperthermia, thereby enhancing tissue penetration and cytotoxicity. This strategy has been investigated in PC as adjuvant treatment after curative resection and as consolidation therapy for limited PM. The key characteristics, efficacy outcomes, and safety profiles of representative clinical studies are summarized in Table 2.

**TABLE 2:** Hyperthermic intraperitoneal chemotherapy: Intraoperative regional chemotherapy with heat-enhanced drug delivery.

Author/Year	Study Type	Regimen	Patient Population	Efficacy Outcomes	Safety/Conversion
Yurttas et al. (2021)	Phase I/II	HIPEC (GEM)	Resected PDAC; postoperative adjuvant; PS 0–1	30-day mortality 0%; 5-year OS not reported	Grade ≥3 complications 0; Pancreatic fistula 23%
Sugarbaker and Stuart (2021)	Phase II	HIPEC (GEM) + NIPEC (GEM)	Resected PDAC; postoperative adjuvant	mOS 29 months; Local/peritoneal recurrence 0% (n = 8)	Grade 3 complication 8%; Port complication 13%
Tentes (2021)	Prospective	HIPEC (GEM)	Resected PDAC; R0 resection; postoperative adjuvant	mOS 17 months; 5-year OS 24%; Locoregional recurrence 10.3%	30-day mortality 5.1%; Major morbidity 28.2%
Grotz et al. (2023)	Prospective	Laparoscopic HIPEC (CDDP + MMC or CDDP + PTX) + open CRS/HIPEC	First-line; low-volume PM (PCI 2); responded to ≥6 months systemic chemo; PS 0–1	mOS from CRS/HIPEC 26 months; 1-, 2-, 3-year OS 76%, 57%, 39%; CC-0 100%	Grade ≥3 complications 44%; 30-day mortality 4.3%
Gudmundsdottir et al. (2023)	Retrospective	CRS/HIPEC vs. systemic chemo alone	First-line; low-volume PM; responded to ≥6 months systemic chemo; PS 0–1	mOS from PM diagnosis 41 vs. 19 months (p = 0.002); CC-0 91%	Major complications 43%; 30-day mortality 4.3%
Tentes et al. (2018)	Retrospective	CRS/HIPEC (GEM or CDDP + MMC)	Peritoneal carcinomatosis from PDAC; salvage	CC-0/1 7/8 procedures; mOS 12–13 months	30-day mortality 33%; Major complications 50%
Yan et al. (2024)	Retrospective	CRS/HIPEC (docetaxel + CDDP)	PM from PDAC; mixed synchronous/metachronous	mOS 24.2 months; CC-0/1 60%	Grade ≥3 complications 20%; 30-day mortality 0%
Padilla-Valverde et al. (2024)	phase II/III	R0 resection + HIPEC (GEM) vs. R0 resection alone	Resected PDAC; postoperative adjuvant	Locoregional recurrence 10% vs. 52% (p = 0.022); mOS 18 vs. 17.1 mo (p = 0.899)	Grade ≥3 complications 14% vs. 29%; 30-day mortality 5% vs. 5%

Abbreviations: HIPEC, hyperthermic intraperitoneal chemotherapy; CRS, cytoreductive surgery; NIPEC, normothermic intraperitoneal chemotherapy; GEM, gemcitabine; CDDP, cisplatin; MMC, mitomycin C; PTX, paclitaxel; PDAC, pancreatic ductal adenocarcinoma; PM, peritoneal metastasis; PCI, peritoneal cancer index; CC, completeness of cytoreduction; mOS, median OS; mo, months; PS, performance status.

The safety of the adjunctive use of HIPEC with pancreatic resection has been prospectively evaluated. The PanHIPEC trial enrolled 16 patients with resected PDAC who received HIPEC with gemcitabine (1,000 mg/m^2^, 42 °C, 60 min). There was no 30-day mortality, and no grade ≥3 HIPEC-related adverse events were observed (Yurttas et al., 2021). A phase II study enrolled 12 patients undergoing pancreaticoduodenectomy plus HIPEC with gemcitabine, followed by six cycles of normothermic intraperitoneal gemcitabine. Among the eight patients who completed the protocol, only one grade 3 complication was reported, with no local or peritoneal recurrences (Sugarbaker and Stuart, 2021). In a series of 39 patients who underwent R0 resection combined with gemcitabine-based HIPEC, the reported outcomes included a 30-day mortality of 5.1%, a major morbidity rate of 28.2%, a 5-year OS of 24%, and a locoregional recurrence rate of 10.3% (Tentes, 2021).

HIPEC is typically combined with cytoreductive surgery (CRS) in a sequential “debulking and hyperthermic perfusion” approach, and this combination has been investigated in highly selected populations. A prospective pilot study enrolled 18 patients with low-volume PM who had responded to ≥6 months of systemic chemotherapy. All achieved complete cytoreduction followed by HIPEC (cisplatin + mitomycin C or cisplatin + paclitaxel). Median OS from CRS/HIPEC was 26 months, with 1-, 2-, and 3-year survival rates of 76%, 57%, and 39%, respectively. Grade ≥3 complications were observed in 44% of patients, including one case of 30-day mortality (Grotz et al., 2023). A matched comparison showed that CRS/HIPEC patients had a median OS of 41 months from diagnosis of PM compared with 19 months for systemic therapy alone (p = 0.002) (Gudmundsdottir et al., 2023). Other retrospective series yielded more modest outcomes. In a cohort of six patients with PC and peritoneal carcinomatosis treated with CRS/HIPEC, the 30-day mortality rate was 33%, and the median OS was 12–13 months (Tentes et al., 2018). A study of 10 patients reported a median OS of 24.2 months after CRS/HIPEC (docetaxel + cisplatin), with two severe adverse events but no perioperative deaths (Yan et al., 2024). A randomized trial of 42 patients compared R0 resection alone with R0 resection combined with gemcitabine-based HIPEC (120 mg/m2, 30 min). HIPEC significantly reduced locoregional recurrence (10% vs. 52%, p = 0.022) and lowered the levels of PC stem cells in peritoneal fluid, although there was no significant difference in OS (Padilla-Valverde et al., 2024).

Three factors consistently governed patient selection across these HIPEC studies: performance status, ascites burden, and response to prior systemic therapy. Most protocols restricted eligibility to patients with an ECOG performance status of 0 or one and/or a Karnofsky score of at least 70%. Patients presenting with low-volume peritoneal disease (typically defined as a Peritoneal Cancer Index <7) and a favorable response to at least 6 months of multiagent chemotherapy demonstrated improved outcomes. In contrast, patients with massive ascites or extensive carcinomatosis were often poor candidates. The 33% 30-day mortality reported by Tentes et al. (2018) in a salvage cohort illustrates the lethal consequence of operating on poorly selected patients. However, the existing evidence remains heterogeneous. The studies comprise prospective phase I/II trials, randomized studies, and small retrospective series. HIPEC regimens exhibited significant variation, ranging from gemcitabine monotherapy to combination protocols such as cisplatin plus mitomycin C or cisplatin plus paclitaxel. Endpoints also differed, including 30-day mortality, OS, and recurrence patterns. This variability precludes direct comparisons and underscores the need for clearer guidelines on the risk-benefit profile of combining pancreatic resection with HIPEC.

### 6.3 Pressurized intraperitoneal aerosol chemotherapy

Pressurized intraperitoneal aerosol chemotherapy (PIPAC) is an innovative regional chemotherapy technique that delivers aerosolized drugs into the peritoneal cavity under controlled pressure (12–15 mmHg). This method improves the uniformity of drug distribution across peritoneal surfaces and enhances tissue penetration depth up to several millimeters, overcoming the limitations of conventional intraperitoneal chemotherapy characterized by heterogeneous drug exposure (Dedrick and Flessner, 1997). Table 3 summarizes the key characteristics, efficacy outcomes, and safety profiles of representative clinical studies.

**TABLE 3:** Pressurized intraperitoneal aerosol chemotherapy: a pressure-controlled aerosol delivery system enhancing homogeneous distribution and tissue penetration.

Author/Year	Study Type	Regimen	Patient Population	Efficacy Outcomes	Safety/Conversion
Graversen et al. (2017)	Prospective	PIPAC C/D	PM from PC; salvage; PS 0–1	Histological regression 80%; mOS 14 months	Grade ≥3 AEs 0
Di Giorgio et al. (2020)	Retrospective	PIPAC C/D or PIPAC Ox	PM from pancreatic or biliary cancer; salvage; PS 0–2	Pathological response 50%; mOS from PIPAC 19.7 months; mOS from PM 16.2 months	Grade ≥3 AEs 0%; Intraoperative perforation 5%
Horvath et al. (2018)	Prospective	PIPAC C/D	PM from pancreatic or biliary cancer; salvage; KI ≥ 60%	Complete regression 33%; Major regression 8%; mOS 12.7 months	Grade ≥3 AEs 0
Khosrawipour et al. (2017)	Retrospective	PIPAC C/D	PM from PC; salvage; KI ≥ 60%	mOS 36.6 weeks; Complete/high-grade regression 35%	Grade ≥3 AEs 0; 30-day mortality 2.4%
Graversen et al. (2023)	phase II	PIPAC C/D or PIPAC Ox	PM from various primaries (incl. pancreatic); PS 0–1	PRGS ≤2 at PIPAC 3 61%; PRGS ≤2 independent prognostic factor; mOS 8.2 months	Grade ≥3 AEs 0%; 30-day mortality 3.6%
Kryh-Jensen et al. (2023)	Retrospective	PIPAC C/D or PIPAC Ox (bidirectional ± systemic chemo)	PM from gastric, pancreatic, or colorectal cancer; PS 0–1	LTS definition 15 months; LTS achieved 63%; Histological response 85%	Bidirectional treatment 37%
Di Giorgio et al. (2024)	Phase II	PIPAC nab-PTX + i.v. GnP	First-line or later; PM from PC; PS 0–1	Primary endpoint DCR; Secondary endpoints PFS, OS, pathological response, QoL	Phase II ongoing; Simon two-stage design
Nielsen et al. (2021)	Prospective	PIPAC C/D	PM from PC; salvage; PS 0–1	Mean PRGS 1.91 to 1.58 (p = 0.02); KRAS mutation in post-PIPAC biopsies 67%	No grade ≥3 AEs

Abbreviations: PIPAC, pressurized intraperitoneal aerosol chemotherapy; C/D, cisplatin + doxorubicin; Ox, oxaliplatin; nab-PTX, nab-paclitaxel; GnP, gemcitabine plus nab-paclitaxel; PM, peritoneal metastasis; PC, pancreatic cancer; PS, performance status; KI, karnofsky index; mOS, median OS; PFS, progression-free survival; DCR, disease control rate; PRGS, peritoneal regression grading score; LTS, long-term survival; AEs, adverse events; mo, months; wk, weeks; QoL, quality of life.

The feasibility and safety of PIPAC in patients with PM from PC have been evaluated in several prospective series. In an early pilot study of five patients with PM from PC, PIPAC with cisplatin and doxorubicin (PIPAC C/D) was well tolerated, with no grade 3 or four adverse events observed, and four of the five patients achieved histological regression according to the Peritoneal Regression Grading Score (PRGS) (Graversen et al., 2017). A larger series of 20 patients with PM from pancreatic adenocarcinoma treated with PIPAC C/D or oxaliplatin (PIPAC Ox) demonstrated a pathological response rate of 50% among those receiving at least two cycles, with a median OS of 9.7 months following the initial PIPAC procedure; no grade 3 or 4 adverse events were observed (Di Giorgio et al., 2020). A subsequent study of 12 patients with PM from pancreatic or biliary tract cancer reported complete tumor regression in four patients and major regression in one patient, with a median OS of 12.7 months among the PC subgroup (Horvath et al., 2018). A retrospective analysis of 20 patients with PM from pancreatic adenocarcinoma treated with PIPAC C/D reported a median OS of 36.6 weeks following the initial PIPAC procedure, with complete or high-grade tumor regression in 35% of patients (Khosrawipour et al., 2017).

The prognostic value of histologic response to PIPAC has been demonstrated in prospective trials. In the prospective PIPAC-OPC2 study, 110 patients with PM from various primary tumors underwent PIPAC C/D or PIPAC Ox. Among patients who completed three PIPAC cycles, 61% achieved a complete or major histological response (PRGS 1–2). The mean PRGS decreased from 2.50 at baseline to 1.79 after the third PIPAC cycle, and a PRGS ≤2 was the only independent prognostic factor for survival in the multivariate analysis (Graversen et al., 2023). A subsequent analysis of long-term survivors from the same cohort, defining long-term survival as 21 months for gastric cancer, 15 months for pancreatic cancer, and 24 months for colorectal cancer, revealed that 85% of patients with pancreatic PM meeting these criteria demonstrated a histological response to treatment (Kryh-Jensen et al., 2023).

The combination of PIPAC with systemic chemotherapy (bidirectional treatment) has also been explored. A phase II trial (Nab-PIPAC) is currently evaluating the combination of systemic nab-paclitaxel plus gemcitabine with intraperitoneal nab-paclitaxel administered via PIPAC in patients with PM from PC, using the disease control rate as the primary endpoint (Di Giorgio et al., 2024). The ability to obtain serial peritoneal biopsies during PIPAC also enables molecular profiling; next-generation sequencing of peritoneal biopsies and lavage fluid has demonstrated that KRAS mutations can be detected in post-PIPAC PM samples from PC at frequencies similar to primary tumors, supporting the utility of this approach for personalized treatment strategies (Nielsen et al., 2021).

PIPAC studies have adopted a distinct strategy by focusing on histological response to guide patient selection. Most series required good performance status, typically ECOG 0 or 1, whereas the presence of high-volume ascites consistently predicted poor outcomes, often precluding patients from completing multiple cycles. Nearly all participants exhibited disease progression following prior palliative chemotherapy; consequently, PIPAC was used primarily as a salvage treatment. The PRGS has demonstrated prognostic utility. In the prospective PIPAC-OPC2 study, a PRGS of or lower after three cycles was the only independent predictor of survival. However, the available evidence remains heterogeneous. The literature comprises prospective trials, retrospective series, and registry analyses employing varied PIPAC regimens (e.g., cisplatin plus doxorubicin, oxaliplatin, or nab-paclitaxel). Furthermore, endpoints vary widely, ranging from histological response to OS, with time-to-event measurements calculated either from the initial PIPAC procedure or from the diagnosis of PM. Future disease-specific trials that validate PRGS as a prognostic tool are needed.

### 6.4 Intraperitoneal chemotherapy combined with immunotherapy

The extensively documented upregulation of PD-L1, engagement of the TIGIT/CD155 axis, and downregulation of MHC-I in PC, as discussed in the preceding sections, would theoretically position immune checkpoint inhibitors (ICIs) as a rational therapeutic strategy. However, the low immunogenicity, marked heterogeneity, dense stroma, and poor vascularity of PDAC impede drug delivery, while activated CAFs recruit immunosuppressive cells. These converging barriers underlie the markedly limited clinical activity of ICIs in unselected patients with metastatic PDAC, including those with peritoneal involvement. The lack of validated predictive biomarkers further constrains the therapeutic potential of conventional first-line chemo-immunotherapy combinations (Hilmi et al., 2023).

Intraperitoneal chemotherapy combined with immunotherapy is a promising strategy under active investigation. Intraperitoneal delivery of modified vaccinia virus Ankara encoding single chain IL-12 (MVA.scIL-12) has shown significant preclinical efficacy in peritoneal carcinomatosis, which is attributed to selective omental targeting and robust antitumor immune induction (Bella et al., 2023). More recently, a 2025 study demonstrated that red blood cell-derived extracellular vesicles co-delivering KRAS antisense oligonucleotides and a RIG-I agonist (immunomodulatory RNA) directly into the peritoneal cavity suppressed tumor growth, reduced metastatic burden, and prolonged survival in preclinical models, while also activating both innate and adaptive immunity. This approach was well tolerated in non-human primates without systemic toxicity (Nguyen et al., 2025). Separately, an ongoing trial (NCT03172416) is evaluating PIPAC in combination with nivolumab in patients with PM, based on the rationale that locoregional chemotherapy can induce immunogenic cell death and prime the microenvironment for immune checkpoint blockade (Kim et al., 2021). These examples illustrate a broader principle: overcoming the profoundly immunosuppressive peritoneal milieu likely requires multimodal strategies that integrate locoregionally induced immunogenic cell death with intraperitoneal immunotherapy. Given the scarcity of such investigations to date, further preclinical and clinical studies are urgently needed to explore additional combinatorial regimens, optimize drug sequencing and dosing, and identify predictive biomarkers.

## 7 Conclusion

PM in PC is orchestrated by an interdependent network of four regulatory hubs: metabolic reprogramming, extracellular vesicles, cancer stem cells, and a dynamically remodeled immune microenvironment. These mechanisms act synergistically to facilitate peritoneal spread and limit treatment efficacy. Emerging locoregional strategies have shown promise in overcoming the peritoneal plasma barrier, yielding outcomes such as cytological conversion, ascites control, and even conversion surgery in highly selected patients. However, the evidence remains heterogeneous due to variable patient selection, differing regimens, and inconsistent endpoints. Critical gaps persist, including the absence of validated biomarkers for patient stratification, the lack of clear criteria for integrating locoregional therapy with systemic or immune-based treatments, and insufficient preclinical models that recapitulate the human peritoneal immune stroma. Addressing these gaps will require multi-omics profiling of patient samples, rigorous biomarker validation, and rationally designed combination trials. Progress in these areas is essential to transform intraperitoneal drug delivery from a palliative option into a precision-guided approach that meaningfully improves outcomes for patients with peritoneal dissemination of PC.

It is important to note that the majority of mechanistic insights discussed in this review are derived from preclinical models, including cell lines and animal studies. Clinical validation of these findings in patients with PM remains limited, and large-scale prospective studies are urgently needed to translate these mechanistic understandings into clinical practice.

## References

[B1] Amrutkar M. Gladhaug I. P. (2021). Stellate cells aid growth-permissive metabolic reprogramming and promote gemcitabine chemoresistance in pancreatic cancer. Cancers 13, 601. 10.3390/cancers13040601 33546284 PMC7913350

[B2] Anand S. Khan M. A. Zubair H. Sudan S. K. Vikramdeo K. S. Deshmukh S. K. (2023). MYB sustains hypoxic survival of pancreatic cancer cells by facilitating metabolic reprogramming. EMBO Rep. 24, e55643. 10.15252/embr.202255643 36592158 PMC9986821

[B3] Araki O. Tsuda M. Omatsu M. Namikawa M. Sono M. Fukunaga Y. (2023). Brg1 controls stemness and metastasis of pancreatic cancer through regulating hypoxia pathway. Oncogene 42, 2139–2152. 10.1038/s41388-023-02716-4 37198398

[B4] Avula L. R. Hagerty B. Alewine C. (2020). Molecular mediators of peritoneal metastasis in pancreatic cancer. Cancer Metastasis Rev. 39, 1223–1243. 10.1007/s10555-020-09924-4 32780245 PMC7680314

[B5] Avula L. R. Rudloff M. El-Behaedi S. Arons D. Albalawy R. Chen X. (2020). Mesothelin enhances tumor vascularity in newly forming pancreatic peritoneal metastases. Mol. Cancer Res. MCR 18, 229–239. 10.1158/1541-7786.MCR-19-0688 31676721 PMC8139242

[B6] Barriga F. M. Tsanov K. M. Ho Y.-J. Sohail N. Zhang A. Baslan T. (2022). MACHETE identifies interferon-encompassing chromosome 9p21.3 deletions as mediators of immune evasion and metastasis. Nat. Cancer 3, 1367–1385. 10.1038/s43018-022-00443-5 36344707 PMC9701143

[B7] Bella Á. Melero I. Berraondo P. Aranda F. (2023). The significance of the omentum in locoregional immunotherapy for peritoneal carcinomatosis. OncoImmunology 12, 2285106. 10.1080/2162402x.2023.2285106 38126032 PMC10732655

[B8] Bose M. Sanders A. De C. Zhou R. Lala P. Shwartz S. (2023). Targeting tumor-associated MUC1 overcomes anoikis-resistance in pancreatic cancer. Transl. Res. J. Lab. Clin. Med. 253, 41–56. 10.1016/j.trsl.2022.08.010 36031050

[B9] Cai Z. Li Y. Ma M. Wang L. Wang H. Liu M. (2023). Adipocytes promote pancreatic cancer migration and invasion through fatty acid metabolic reprogramming. Oncol. Rep. 50, 141. 10.3892/or.2023.8578 37264956 PMC10285608

[B10] Canel M. Sławińska A. D. Lonergan D. W. Kallor A. A. Upstill-Goddard R. Davidson C. (2023). FAK suppresses antigen processing and presentation to promote immune evasion in pancreatic cancer. Gut 73, 131–155. 10.1136/gutjnl-2022-327927 36977556 PMC10715489

[B11] Ceelen W. Braet H. Van Ramshorst G. Willaert W. Remaut K. (2020). Intraperitoneal chemotherapy for peritoneal metastases: an expert opinion. Expert Opin. Drug Deliv. 17, 511–522. 10.1080/17425247.2020.1736551 32142389

[B12] Chakrabarti S. Kamgar M. Mahipal A. (2022). Systemic therapy of metastatic pancreatic adenocarcinoma: current status, challenges, and opportunities. Cancers 14, 2588. 10.3390/cancers14112588 35681565 PMC9179239

[B13] Chang J. Li H. Zhu Z. Mei P. Hu W. Xiong X. (2022). microRNA-21-5p from M2 macrophage-derived extracellular vesicles promotes the differentiation and activity of pancreatic cancer stem cells by mediating KLF3. Cell Biol. Toxicol. 38, 577–590. 10.1007/s10565-021-09597-x 33728488 PMC9343318

[B14] Chen X. Zhou Z. Xie L. Qiao K. Jia Y. Liu S. (2025). Single-cell resolution spatial analysis of antigen-presenting cancer-associated fibroblast niches. Cancer Cell 43, 2224–2240.e9. 10.1016/j.ccell.2025.09.001 41005303 PMC12479095

[B15] Chiba K. Hata T. Mizuma M. Masuda K. Aoki S. Takadate T. (2022). Impact of tumor-derived DNA testing in peritoneal lavage of pancreatic cancer patients with and without occult intra-abdominal metastases. Ann. Surg. Oncol. 29, 2685–2697. 10.1245/s10434-021-10997-w 34739641

[B16] Contreras C. M. Stanelle E. J. Mansour J. Hinshaw J. L. Rikkers L. F. Rettammel R. (2009). Staging laparoscopy enhances the detection of occult metastases in patients with pancreatic adenocarcinoma. J. Surg. Oncol. 100, 663–669. 10.1002/jso.21402 19780095

[B17] De R. A. Cameron I. C. Gomez D. (2016). Indications for staging laparoscopy in pancreatic cancer. Hpb 18, 13–20. 10.1016/j.hpb.2015.10.004 26776846 PMC4750228

[B18] Dedrick R. L. Flessner M. F. (1997). Pharmacokinetic problems in peritoneal drug administration: tissue penetration and surface exposure. J. Natl. Cancer Inst. 89, 480–487. 10.1093/jnci/89.7.480 9086004

[B19] Deng Y. Xia X. Zhao Y. Zhao Z. Martinez C. Yin W. (2021). Glucocorticoid receptor regulates PD-L1 and MHC-I in pancreatic cancer cells to promote immune evasion and immunotherapy resistance. Nat. Commun. 12, 7041. 10.1038/s41467-021-27349-7 34873175 PMC8649069

[B20] Di Giorgio A. Sgarbura O. Rotolo S. Schena C. A. Bagalà C. Inzani F. (2020). Pressurized intraperitoneal aerosol chemotherapy with cisplatin and doxorubicin or oxaliplatin for peritoneal metastasis from pancreatic adenocarcinoma and cholangiocarcinoma. Ther. Adv. Med. Oncol. 12, 1758835920940887. 10.1177/1758835920940887 32782488 PMC7383654

[B21] Di Giorgio A. Ferracci F. Bagalà C. Carbone C. Salvatore L. Strippoli A. (2024). Combined nabpaclitaxel pressurized intraPeritoneal aerosol chemotherapy with systemic nabpaclitaxel-gemcitabine chemotherapy for pancreatic cancer peritoneal metastases: protocol of single-arm, open-label, phase II trial (Nab-PIPAC trial). Pleura Perit. 9, 121–129. 10.1515/pp-2024-0010 PMC11558173 39544430

[B22] Ernesti-Soldatkin A. Neu C. T. Heydel B. Krannich F. Laumen H. Gutschner T. (2026). FOXM1 regulates platelet-induced anoikis resistance in pancreatic cancer cells. Cell Commun. Signal CCS 24, 53. 10.1186/s12964-025-02644-8 41535944 PMC12849465

[B23] Ewa T. Panchwagh N. Tai C.-H. Avula L. R. Joseph S. Rudloff M. W. (2024). Excess shed mesothelin disrupts pancreatic cancer cell clustering to impair peritoneal colonization. FASEB J. Off. Publ. Fed. Am. Soc. Exp. Biol. 38, e70247. 10.1096/fj.202400446r PMC11646052 39673668

[B24] Fang Z. Bunston C. Xu Y. Ruge F. Sui L. Liu M. (2024). Implication of capillary morphogenesis gene 2 (CMG2) in the disease progression and peritoneal metastasis of pancreatic cancer. Cancers 16, 2893. 10.3390/cancers16162893 39199664 PMC11352480

[B25] Freed-Pastor W. A. Lambert L. J. Ely Z. A. Pattada N. B. Bhutkar A. Eng G. (2021). The CD155/TIGIT axis promotes and maintains immune evasion in neoantigen-expressing pancreatic cancer. Cancer Cell 39, 1342–1360.e14. 10.1016/j.ccell.2021.07.007 34358448 PMC8511341

[B26] Gaballah A. H. Algazzar M. Kazi I. A. Badawy M. Guys N. P. Mohamed E. A. S. (2024). The peritoneum: anatomy, pathologic findings, and patterns of disease spread. Radiogr. Rev. Publ. Radiol. Soc. N. Am. Inc. 44, e230216. 10.1148/rg.230216 39088361

[B27] Ganatra N. Abdelhakeem A. Jain P. Kamatham S. Elantably D. Adeoye O. (2026). Intraperitoneal chemotherapy strategies in pancreatic ductal adenocarcinoma: a systematic review of hyperthermic intraperitoneal chemotherapy, normothermic intraperitoneal chemotherapy, and pressurized intraperitoneal aerosol chemotherapy. Cancers 18, 182. 10.3390/cancers18020182 41595106 PMC12838573

[B28] Gao F. Sun K. Wang S. Zhang X. Bai X. (2025). Lactate metabolism reprogramming in PDAC: potential for tumor therapy. Biochim. Biophys. Acta Rev. Cancer 1880, 189373. 10.1016/j.bbcan.2025.189373 40513632

[B29] Graversen M. Detlefsen S. Bjerregaard J. K. Pfeiffer P. Mortensen M. B. (2017). Peritoneal metastasis from pancreatic cancer treated with pressurized intraperitoneal aerosol chemotherapy (PIPAC). Clin. Exp. Metastasis 34, 309–314. 10.1007/s10585-017-9849-7 28516306

[B30] Graversen M. Detlefsen S. Ainsworth A. P. Fristrup C. W. Knudsen A. O. Pfeiffer P. (2023). Treatment of peritoneal metastasis with pressurized intraperitoneal aerosol chemotherapy: results from the prospective PIPAC-OPC2 study. Ann. Surg. Oncol. 30, 2634–2644. 10.1245/s10434-022-13010-0 36602663

[B31] Gromisch C. M. Tan G. L. A. Pasion K. A. Moran A.-M. Gromisch M. S. Grinstaff M. W. (2021). Humanized anti-DEspR IgG4S228P antibody increases overall survival in a pancreatic cancer stem cell-xenograft peritoneal carcinomatosis ratnu/nu model. BMC Cancer 21, 407. 10.1186/s12885-021-08107-w 33853558 PMC8048286

[B32] Grotz T. E. Yonkus J. A. Thiels C. A. Warner S. G. McWilliams R. R. Mahipal A. (2023). Cytoreduction with hyperthermic intraperitoneal chemoperfusion for pancreatic cancer with low-volume peritoneal metastasis: results from a prospective pilot study. Ann. Surg. Oncol. 30, 395–403. 10.1245/s10434-022-12328-z 35972667

[B33] Gudmundsdottir H. Yonkus J. A. Thiels C. A. Warner S. G. Cleary S. P. Kendrick M. L. (2023). Oncologic outcomes of cytoreductive surgery and hyperthermic intraperitoneal chemotherapy for highly selected patients with metastatic pancreatic ductal adenocarcinoma. Ann. Surg. Oncol. 30, 7833–7839. 10.1245/s10434-023-14138-3 37596449

[B34] Gupta V. K. Sharma N. S. Durden B. Garrido V. T. Kesh K. Edwards D. (2021). Hypoxia-driven oncometabolite L-2HG maintains stemness-differentiation balance and facilitates immune evasion in pancreatic cancer. Cancer Res. 81, 4001–4013. 10.1158/0008-5472.can-20-2562 33990397 PMC8338764

[B35] Han L. Zhang D. Xiao W. Zeng C. Chen Y. (2026). PIK3CG deficiency promotes metabolic reprogramming in pancreatic cancer by suppressing GLS2-driven glutamine metabolism. Int. Immunopharmacol. 172, 116106. 10.1016/j.intimp.2025.116106 41539001

[B36] Hata T. Mizuma M. Iseki M. Takadate T. Ishida M. Nakagawa K. (2021). Circulating tumor DNA as a predictive marker for occult metastases in pancreatic cancer patients with radiographically non-metastatic disease. J. Hepato-Biliary-Pancreat Sci. 28, 648–658. 10.1002/jhbp.993 34022116

[B37] Hilmi M. Delaye M. Muzzolini M. Nicolle R. Cros J. Hammel P. (2023). The immunological landscape in pancreatic ductal adenocarcinoma and overcoming resistance to immunotherapy. Lancet Gastroenterol. Hepatol. 8, 1129–1142. 10.1016/s2468-1253(23)00207-8 37866368

[B38] Horvath P. Beckert S. Struller F. Königsrainer A. Reymond M. A. (2018). Pressurized intraperitoneal aerosol chemotherapy (PIPAC) for peritoneal metastases of pancreas and biliary tract cancer. Clin. Exp. Metastasis 35, 635–640. 10.1007/s10585-018-9925-7 30062506

[B39] Hu J. Wang J. Guo X. Fan Q. Li X. Li K. (2024). MSLN induced EMT, cancer stem cell traits and chemotherapy resistance of pancreatic cancer cells. Heliyon 10, e29210. 10.1016/j.heliyon.2024.e29210 38628720 PMC11019237

[B40] Inzunza J. Del Valle A. C. (2025). Deciphering the liver’s role in pancreatic cancer metastasis: pathways and therapeutic approaches. Npj Precis. Oncol. 9, 395–403. 10.1038/s41698-025-01202-2 41331510 PMC12678447

[B41] Ischenko I. D’Amico S. Rao M. Li J. Hayman M. J. Powers S. (2021). KRAS drives immune evasion in a genetic model of pancreatic cancer. Nat. Commun. 12, 1482. 10.1038/s41467-021-21736-w 33674596 PMC7935870

[B42] Jambor M. A. Ashrafizadeh A. Nahm C. B. Clarke S. J. Pavlakis N. Kneebone A. (2022). The role of staging laparoscopy in pancreatic adenocarcinoma and its effect on patients’ survival. World J. Surg. Oncol. 20, 337. 10.1186/s12957-022-02803-y 36217193 PMC9552432

[B43] Jeong J.-Y. Kim T.-B. Kim J. Choi H. W. Kim E. J. Yoo H. J. (2020). Diversity in the extracellular vesicle-derived microbiome of tissues according to tumor progression in pancreatic cancer. Cancers 12, 2346. 10.3390/cancers12092346 32825137 PMC7563179

[B44] Jin W. Yin H. Li H. Yu X.-J. Xu H.-X. Liu L. (2021). Neutrophil extracellular DNA traps promote pancreatic cancer cells migration and invasion by activating EGFR/ERK pathway. J. Cell Mol. Med. 25, 5443–5456. 10.1111/jcmm.16555 33955688 PMC8184670

[B45] Jinno N. Yoshida M. Hayashi K. Naitoh I. Hori Y. Natsume M. (2021). Autotaxin in ascites promotes peritoneal dissemination in pancreatic cancer. Cancer Sci. 112, 668–678. 10.1111/cas.14689 33053268 PMC7893983

[B46] Kamakura M. Uehara T. Iwaya M. Asaka S. Kobayashi S. Nakajima T. (2022). LGR5 expression and clinicopathological features of the invasive front in the fat infiltration area of pancreatic cancer. Diagn Pathol. 17, 21. 10.1186/s13000-022-01203-w 35123536 PMC8818226

[B47] Kang H. W. Kim J. H. Jeong J. W. Fang S. Yun W.-G. Jung H.-S. (2025). SLC6A14-mediated glutamine promotes SYTL4-CXCL8 axis activation to drive gemcitabine resistance and immune evasion in pancreatic cancer. Exp. Mol. Med. 57, 2943–2956. 10.1038/s12276-025-01596-w 41444422 PMC12800172

[B48] Kaşıkcı E. Aydemir E. Bayrak Ö. F. Şahin F. (2020). Inhibition of migration, invasion and drug resistance of pancreatic adenocarcinoma cells - role of snail, slug and twist and small molecule inhibitors. OncoTargets Ther. 13, 5763–5777. 10.2147/ott.s253418 PMC7308789 32606788

[B49] Kemper M. Schiecke A. Maar H. Nikulin S. Poloznikov A. Galatenko V. (2021). Integrin alpha-V is an important driver in pancreatic adenocarcinoma progression. J. Exp. Clin. Cancer Res. CR 40, 214. 10.1186/s13046-021-01946-2 34174926 PMC8235815

[B50] Khosrawipour T. Khosrawipour V. Giger-Pabst U. (2017). Pressurized intra peritoneal aerosol chemotherapy in patients suffering from peritoneal carcinomatosis of pancreatic adenocarcinoma. PLOS One 12, e0186709. 10.1371/journal.pone.0186709 29049340 PMC5648228

[B51] Kim G. Tan H. L. Sundar R. Lieske B. Chee C. E. Ho J. (2021). PIPAC-OX: a phase i study of oxaliplatin-based pressurized intraperitoneal aerosol chemotherapy in patients with peritoneal metastases. Clin Cancer Res. 27(7):1875-1881. 10.1158/1078-0432.CCR-20-2152 33148667

[B52] Kim D. Kaushal D. Wilson R. B. (2026). Metastatic organotropism in peritoneal metastasis: paget’s hypothesis revisited. Clin. Exp. Med. 26, 123. 10.1007/s10238-025-01868-9 41609906 PMC12858513

[B53] Kitayama H. Tsuji Y. Kondo T. Sugiyama J. Hirayama M. Yamamoto K. (2016). Conversion therapy for pancreatic cancer with peritoneal metastases using intravenous and intraperitoneal paclitaxel with S-1. Mol. Clin. Oncol. 5, 779–782. 10.3892/mco.2016.1051 28105356 PMC5228476

[B54] Kryh-Jensen C. G. Fristrup C. W. Ainsworth A. P. Detlefsen S. Mortensen M. B. Pfeiffer P. (2023). What is long-term Survival in Patients with Peritoneal Metastasis from Gastric, Pancreatic, or Colorectal Cancer? A study of Patients Treated with Systemic Chemotherapy and Pressurized Intraperitoneal Aerosol Chemotherapy (PIPAC), 8. Berlin, Germany: De Gruyter, 147–155. 38144215 10.1515/pp-2023-0038 PMC10739291

[B55] Lakshmanan I. Marimuthu S. Chaudhary S. Seshacharyulu P. Rachagani S. Muniyan S. (2022). Muc16 depletion diminishes KRAS-induced tumorigenesis and metastasis by altering tumor microenvironment factors in pancreatic ductal adenocarcinoma. Oncogene 41, 5147–5159. 10.1038/s41388-022-02493-6 36271032 PMC9841597

[B56] Lao M. Zhang X. Li Z. Sun K. Yang H. Wang S. (2025). Lipid metabolism reprograming by SREBP1-PCSK9 targeting sensitizes pancreatic cancer to immunochemotherapy. Cancer Commun. 45, 1011–1037. 10.1002/cac2.70038 PMC12766276 40439109

[B57] Li J. Guo T. (2022). Role of peritoneal mesothelial cells in the progression of peritoneal metastases. Cancers 14, 2856. 10.3390/cancers14122856 35740521 PMC9221366

[B58] Li T. Guo T. Liu H. Jiang H. Wang Y. (2021). Platelet-derived growth factor-BB mediates pancreatic cancer malignancy via regulation of the hippo/yes-associated protein signaling pathway. Oncol. Rep. 45, 83–94. 10.3892/or.2020.7859 PMC7709832 33416116

[B59] Li X. Zhang Y. Yan Z. Jiang W. Rui S. (2024). Global, regional and national burden of pancreatic cancer and its attributable risk factors from 2019 to 2021, with projection to 2044. Front. Oncol. 14, 1521788. 10.3389/fonc.2024.1521788 39876895 PMC11772166

[B60] Li X. Yu T. Yu Z. Zou X. He J. Wang D. (2025). BNC2 as a novel driver of pancreatic cancer progression through transcriptional regulation of COL3A1 and epithelial-to-mesenchymal transition. Med. Oncol. 43, 11. 10.1007/s12032-025-03139-9 41269436

[B61] Liu M. Silva-Sanchez A. Randall T. D. Meza-Perez S. (2021). Specialized immune responses in the peritoneal cavity and omentum. J. Leukoc. Biol. 109, 717–729. 10.1002/jlb.5mir0720-271rr 32881077 PMC7921210

[B62] Lu X. Wu Y. Cao R. Yu X. Gong J. (2022). CXCL12 secreted by pancreatic stellate cells accelerates gemcitabine resistance of pancreatic cancer by enhancing glycolytic reprogramming. Anim. Cells Syst. 26, 148–157. 10.1080/19768354.2022.2091019 PMC9423839 36046033

[B63] Maloney S. Clarke S. J. Sahni S. Hudson A. Colvin E. Mittal A. (2023). The role of diagnostic, prognostic, and predictive biomarkers in the management of early pancreatic cancer. J. Cancer Res. Clin. Oncol. 149, 13437–13450. 10.1007/s00432-023-05149-4 37460806 PMC10587199

[B64] Meguro Y. Yamaguchi H. Sasanuma H. Shimodaira K. Aoki Y. Chinen T. (2024). Combined intraperitoneal paclitaxel and systemic chemotherapy for patients with massive malignant ascites secondary to pancreatic cancer: a report of two patients. Intern Med. Tokyo Jpn. 63, 2015–2021. 10.2169/internalmedicine.2191-23 PMC11309862 38044154

[B65] Nguyen T. T. T. Dang X. T. T. Phung C. D. Tran L. T. N. Do N. T. P. Yeo E. Y. (2025). Safety and efficacy of KRAS antisense oligonucleotides and RIG-I agonists delivered by extracellular vesicles for pancreatic cancer peritoneal metastasis treatment. J. Control Release Off. J. Control Release Soc. 387, 114239. 10.1016/j.jconrel.2025.114239 40967408

[B66] Nielsen M. Graversen M. Ellebæk S. B. Kristensen T. K. Fristrup C. Pfeiffer P. (2021). Next-generation sequencing and histological response assessment in peritoneal metastasis from pancreatic cancer treated with PIPAC. J. Clin. Pathol. 74, 19–24. 10.1136/jclinpath-2020-206607 32385139 PMC7788484

[B67] Novizio N. Belvedere R. Pessolano E. Tosco A. Porta A. Perretti M. (2020). Annexin A1 released in extracellular vesicles by pancreatic cancer cells activates components of the tumor microenvironment, through interaction with the formyl-peptide receptors. Cells 9, 2719. 10.3390/cells9122719 33353163 PMC7767312

[B68] Nwosu Z. C. Ward M. H. Sajjakulnukit P. Poudel P. Ragulan C. Kasperek S. (2023). Uridine-derived ribose fuels glucose-restricted pancreatic cancer. Nature 618, 151–158. 10.1038/s41586-023-06073-w 37198494 PMC10232363

[B69] Odagiri T. Asano Y. Kagiya T. Matsusaki M. Akashi M. Shimoda H. (2021). The cell line-dependent diversity in initial morphological dynamics of pancreatic cancer cell peritoneal metastasis visualized by an artificial human peritoneal model. J. Surg. Res. 261, 351–360. 10.1016/j.jss.2020.12.046 33493887

[B70] Okabe Y. (2021). Immune niche within the peritoneal cavity. Curr. Top. Microbiol. Immunol. 434, 123–134. 10.1007/978-3-030-86016-5_6 34850285

[B71] Padilla-Valverde D. Bodoque-Villar R. García-Santos E. Sanchez S. Manzanares-Campillo C. Rodriguez M. (2024). Safety and effectiveness of perioperative hyperthermic intraperitoneal chemotherapy with gemcitabine in patients with resected pancreatic ductal adenocarcinoma: clinical trial EudraCT 2016-004298-41. Cancers 16, 1718. 10.3390/cancers16091718 38730669 PMC11083892

[B72] Pauls M. AlJassim AlShareef A. Cheung W. Y. Goodwin R. A. Meyers B. M. Kim C. (2020). Clinical outcomes of pancreas cancer patients with peritoneal spread: results from the chord consortium. J. Clin. Oncol. 38, e19268. 10.1200/jco.2020.38.15_suppl.e19268

[B73] Philipp L.-M. Yesilyurt U.-U. Surrow A. Künstner A. Mehdorn A. S. Hauser C. (2024). Epithelial and mesenchymal-like pancreatic cancer cells exhibit different stem cell phenotypes associated with different metastatic propensities. Cancers 16, 686. 10.3390/cancers16040686 38398077 PMC10886860

[B74] Pisuchpen N. Lennartz S. Parakh A. Kongboonvijit S. Srinivas Rao S. Pierce T. T. (2024). Material density dual-energy CT images: value added in early diagnosis of peritoneal carcinomatosis. Abdom. Radiol. 49, 3496–3506. 10.1007/s00261-024-04451-0 38916617

[B75] Purohit A. Saxena S. Varney M. Prajapati D. R. Kozel J. A. Lazenby A. (2021). Host Cxcr2-dependent regulation of pancreatic cancer growth, angiogenesis, and metastasis. Am. J. Pathol. 191, 759–771. 10.1016/j.ajpath.2021.01.002 33453178 PMC8027924

[B76] Qin C. Zhao B. Wang Y. Li Z. Li T. Zhao Y. (2024). Extracellular vesicles miR-31-5p promotes pancreatic cancer chemoresistance via regulating LATS2-hippo pathway and promoting SPARC secretion from pancreatic stellate cells. J. Extracell. Vesicles 13, e12488. 10.1002/jev2.12488 39104296 PMC11300957

[B77] Ramos C. Gerakopoulos V. Oehler R. (2024). Metastasis-associated fibroblasts in peritoneal surface malignancies. Br. J. Cancer 131, 407–419. 10.1038/s41416-024-02717-4 38783165 PMC11300623

[B78] Romano R. Picca A. Eusebi L. H. U. Marzetti E. Calvani R. Moro L. (2021). Extracellular vesicles and pancreatic cancer: insights on the roles of miRNA, lncRNA, and protein cargos in cancer progression. Cells 10, 1361. 10.3390/cells10061361 34205944 PMC8226820

[B79] Sakaue T. Koga H. Iwamoto H. Nakamura T. Ikezono Y. Abe M. (2019). Glycosylation of ascites-derived exosomal CD133: a potential prognostic biomarker in patients with advanced pancreatic cancer. Med. Mol. Morphol. 52, 198–208. 10.1007/s00795-019-00218-5 30805710

[B80] Sato M. Matsumoto M. Saiki Y. Alam M. Nishizawa H. Rokugo M. (2020). BACH1 promotes pancreatic cancer metastasis by repressing epithelial genes and enhancing epithelial-mesenchymal transition. Cancer Res. 80, 1279–1292. 10.1158/0008-5472.CAN-18-4099 31919242

[B81] Satoi S. Fujii T. Yanagimoto H. Motoi F. Kurata M. Takahara N. (2017). Multicenter phase II study of intravenous and intraperitoneal paclitaxel with S-1 for pancreatic ductal adenocarcinoma patients with peritoneal metastasis. Ann. Surg. 265, 397–401. 10.1097/SLA.0000000000001705 28059968

[B82] Satoi S. Yanagimoto H. Yamamoto T. Hirooka S. Yamaki S. Kosaka H. (2017). Survival benefit of intravenous and intraperitoneal paclitaxel with S-1 in pancreatic ductal adenocarcinoma patients with peritoneal metastasis: a retrospective study in a single institution. J. Hepato-Biliary-Pancreat Sci. 24, 289–296. 10.1002/jhbp.447 28301088

[B83] Satoi S. Takahara N. Fujii T. Isayama H. Yamada S. Tsuji Y. (2022). Synopsis of a clinical practice guideline for pancreatic ductal adenocarcinoma with peritoneal dissemination in Japan; Japan peritoneal malignancy study group. J. Hepato-Biliary-Pancreat Sci. 29, 600–608. 10.1002/jhbp.1085 PMC9306579 34855287

[B84] Sedlacek A. L. Gerber S. A. Randall T. D. van Rooijen N. Frelinger J. G. Lord E. M. (2013). Generation of a dual-functioning antitumor immune response in the peritoneal cavity. Am. J. Pathol. 183, 1318–1328. 10.1016/j.ajpath.2013.06.030 23933065 PMC3791689

[B85] Shukla A. Kalayarasan R. Sai Krishna P. Pottakkat B. (2025). Remnant pancreatic carcinoma: the current status. World J. Clin. Oncol. 16, 107039. 10.5306/wjco.v16.i5.107039 40503403 PMC12149834

[B86] Stefanidis D. Grove K. D. Schwesinger W. H. Thomas C. R. (2006). The current role of staging laparoscopy for adenocarcinoma of the pancreas: a review. Ann. Oncol. 17, 189–199. 10.1093/annonc/mdj013 16236756

[B87] Stoop T. F. Javed A. A. Oba A. Koerkamp B. G. Seufferlein T. Wilmink J. W. (2025). Pancreatic cancer. Lancet Lond Engl. 405, 1182–1202. 10.1016/s0140-6736(25)00261-2 40187844

[B88] Suenaga M. Fujii T. Yamada S. Hayashi M. Shinjo K. Takami H. (2021). Peritoneal lavage tumor DNA as a novel biomarker for predicting peritoneal recurrence in pancreatic ductal adenocarcinoma. Ann. Surg. Oncol. 28, 2277–2286. 10.1245/s10434-020-08990-w 32875467

[B89] Sugarbaker P. H. Stuart O. A. (2021). Intraperitoneal gemcitabine chemotherapy is safe for patients with resected pancreatic cancer: final clinical and pharmacologic data from a phase II protocol and recommended future directions. J. Gastrointest. Oncol. 12, S99–S109. 10.21037/jgo-2020-02 33968430 PMC8100730

[B90] Sun K. Zhang X. Shi J. Huang J. Wang S. Li X. (2025). Elevated protein lactylation promotes immunosuppressive microenvironment and therapeutic resistance in pancreatic ductal adenocarcinoma. J. Clin. Invest. 135, e187024. 10.1172/jci187024 39883522 PMC11957693

[B91] Takahara N. Isayama H. Nakai Y. Ishigami H. Satoi S. Mizuno S. (2016). Intravenous and intraperitoneal paclitaxel with S-1 for treatment of refractory pancreatic cancer with malignant ascites. Invest. New Drugs 34, 636–642. 10.1007/s10637-016-0369-0 27339809

[B92] Takahara N. Nakai Y. Ishigami H. Saito K. Sato T. Hakuta R. (2021). A phase I study of intraperitoneal paclitaxel combined with gemcitabine plus nab-paclitaxel for pancreatic cancer with peritoneal metastasis. Invest. New Drugs 39, 175–181. 10.1007/s10637-020-00982-7 32772340

[B93] Takeda T. Sasaki T. Mie T. Furukawa T. Yamada Y. Kasuga A. (2021). Improved prognosis of pancreatic cancer patients with peritoneal metastasis. Pancreatol. Off. J. Int. Assoc. Pancreatol. IAP Al 21, 903–911. 10.1016/j.pan.2021.03.006 33766484

[B94] Tanji Y. Shimada S. Kato M. Akiyama Y. Hatano M. Tsukihara S. (2026). Cytokine mRNA-based therapy alleviates dendritic cell and T cell paucity to eliminate aggressive pancreatic cancer in preclinical mouse models. EBioMedicine 125, 106137. 10.1016/j.ebiom.2026.106137 41638924 PMC12988560

[B95] Tentes A.-A. K. (2021). Hyperthermic intra-operative intraperitoneal chemotherapy as an adjuvant to pancreatic cancer resection. J. Gastrointest. Oncol. 12, S91–S98. 10.21037/jgo-20-46 33968429 PMC8100705

[B96] Tentes A.-A. Pallas N. Karamveri C. Kyziridis D. Hristakis C. (2018). Cytoreduction and HIPEC for peritoneal carcinomatosis of pancreatic cancer. J. BUON Off. J. Balk. Union Oncol. 23, 482–487. 29745096

[B97] Tomizawa S. Takano S. Eto R. Takayashiki T. Kuboki S. Ohtsuka M. (2023). Semaphorin 3 C enhances putative cancer stemness and accelerates peritoneal dissemination in pancreatic cancer. Cancer Cell Int. 23, 155. 10.1186/s12935-023-03008-3 37537633 PMC10401755

[B98] Wang S. Gao Y. (2021). Pancreatic cancer cell-derived microRNA-155-5p-containing extracellular vesicles promote immune evasion by triggering EHF-dependent activation of akt/NF-κB signaling pathway. Int. Immunopharmacol. 100, 107990. 10.1016/j.intimp.2021.107990 34482266

[B99] Wang C.-A. Chang I.-H. Hou P.-C. Tai Y. J. Li W. N. Hsu P. L. (2020). DUSP2 regulates extracellular vesicle-VEGF-C secretion and pancreatic cancer early dissemination. J. Extracell. Vesicles 9, 1746529. 10.1080/20013078.2020.1746529 32341770 PMC7170376

[B100] Wiedmann L. De Angelis Rigotti F. Vaquero-Siguero N. Donato E. Espinet E. Moll I. (2023). HAPLN1 potentiates peritoneal metastasis in pancreatic cancer. Nat. Commun. 14, 2353. 10.1038/s41467-023-38064-w 37095087 PMC10126109

[B101] Wu M. Tan X. Liu P. Yang Y. Huang Y. Liu X. (2020). Role of exosomal microRNA-125b-5p in conferring the metastatic phenotype among pancreatic cancer cells with different potential of metastasis. Life Sci. 255, 117857. 10.1016/j.lfs.2020.117857 32470446

[B102] Wu D. Chen W. Yang Y. Qin Y. Zu G. Zhang Y. (2023). PITX2 in pancreatic stellate cells promotes EMT in pancreatic cancer cells via the wnt/β-catenin pathway. Acta Biochim. Biophys. Sin. 55, 1393–1403. 10.3724/abbs.2023118 37337632 PMC10520469

[B103] Wu G. Standring O. J. King D. A. Gholami S. Devoe C. E. Thiels C. A. (2025). Management of peritoneal metastasis in patients with pancreatic ductal adenocarcinoma. Curr. Oncol. 32, 103. 10.3390/curroncol32020103 39996904 PMC11854847

[B104] Xie Y. Hang Y. Wang Y. Sleightholm R. Prajapati D. R. Bader J. (2020). Stromal modulation and treatment of metastatic pancreatic cancer with local intraperitoneal triple miRNA/siRNA nanotherapy. ACS Nano 14, 255–271. 10.1021/acsnano.9b03978 31927946 PMC7041410

[B105] Yamada S. Fujii T. Yamamoto T. Takami H. Yoshioka I. Yamaki S. (2020). Phase I/II study of adding intraperitoneal paclitaxel in patients with pancreatic cancer and peritoneal metastasis. Br. J. Surg. 107, 1811–1817. 10.1002/bjs.11792 32638367 PMC7689756

[B106] Yamada S. Fujii T. Yamamoto T. Takami H. Yoshioka I. Yamaki S. (2021). Conversion surgery in patients with pancreatic cancer and peritoneal metastasis. J. Gastrointest. Oncol. 12, S110–S117. 10.21037/jgo-20-243 33968431 PMC8100706

[B107] Yamamoto K. Venida A. Perera R. M. Kimmelman A. C. (2020). Selective autophagy of MHC-I promotes immune evasion of pancreatic cancer. Autophagy 16, 1524–1525. 10.1080/15548627.2020.1769973 32459143 PMC7469632

[B108] Yamamoto T. Satoi S. Yamaki S. Hashimoto D. Ishida M. Ikeura T. (2022). Intraperitoneal paclitaxel treatment for patients with pancreatic ductal adenocarcinoma with peritoneal dissemination provides a survival benefit. Cancers 14, 1354. 10.3390/cancers14051354 35267661 PMC8909716

[B109] Yan G. Zhang K. Yan L. Zhang Y. (2024). Efficacy and safety of cytoreductive surgery combined with hyperthermic intraperitoneal chemotherapy in patients with pancreatic cancer peritoneal metastasis. World J. Surg. Oncol. 22, 212. 10.1186/s12957-024-03464-9 39218891 PMC11367765

[B110] Yang Y. M. Ye L. Ruge F. Fang Z. Ji K. Sanders A. J. (2023). Activated leukocyte cell adhesion molecule (ALCAM), a potential “seed” and “soil” receptor in the peritoneal metastasis of gastrointestinal cancers. Int. J. Mol. Sci. 24, 876. 10.3390/ijms24010876 36614319 PMC9821744

[B111] Yang S. Tang W. Azizian A. Gaedcke J. Ohara Y. Cawley H. (2024). MIF/NR3C2 axis regulates glucose metabolism reprogramming in pancreatic cancer through MAPK-ERK and AP-1 pathways. Carcinogenesis 45, 582–594. 10.1093/carcin/bgae025 38629149 PMC11317528

[B112] Yang Z. Zhang Z. Song Y. Dai C. Zhang W. Zhao D. (2025). PCDH1 facilitates migration, proliferation, and stemness of pancreatic cancer cells through PI3K-akt signaling. Front. Oncol. 15, 1696695. 10.3389/fonc.2025.1696695 41602375 PMC12832269

[B113] Yao H. Huang C. Zou J. Liang W. Zhao Y. Yang K. (2024). Extracellular vesicle-packaged lncRNA from cancer-associated fibroblasts promotes immune evasion by downregulating HLA-a in pancreatic cancer. J. Extracell. Vesicles 13, e12484. 10.1002/jev2.12484 39041344 PMC11263977

[B114] Yeo M. Cho I. R. Lee S. H. Kang H. Jang E. S. Ahn J. (2025). Characteristics of pancreatic cancer-associated ascites and redefined criteria for bacterial peritonitis. Pancreas 55, e471–e480. 10.1097/mpa.0000000000002606 41396838

[B115] Yu B. Gu D. Zhang X. Liu B. Xie J. (2022). Regulation of pancreatic cancer metastasis through the Gli2-YAP1 axis via regulation of anoikis. Genes Dis. 9, 1427–1430. 10.1016/j.gendis.2022.05.010 36157479 PMC9485280

[B116] Yurttas C. Horvath P. Fischer I. Meisner C. Nadalin S. Königsrainer I. (2021). A prospective, phase I/II, open-label pilot trial to assess the safety of hyperthermic intraperitoneal chemotherapy after oncological resection of pancreatic adenocarcinoma. Ann. Surg. Oncol. 28, 9086–9095. 10.1245/s10434-021-10187-8 34131821 PMC8205203

[B117] Zhang L. Sanagapalli S. Stoita A. (2018). Challenges in diagnosis of pancreatic cancer. World J. Gastroenterol. 24, 2047–2060. 10.3748/wjg.v24.i19.2047 29785074 PMC5960811

[B118] Zhang J. Li X. Lu Y. Wang G. Ma Y. (2023). Anoikis-related gene signature for prognostication of pancreatic adenocarcinoma: a multi-omics exploration and verification study. Cancers 15, 3146. 10.3390/cancers15123146 37370756 PMC10296373

[B119] Zhang R. Peng J. Zhang Y. Zheng K. Chen Y. Liu L. (2024). Pancreatic cancer cell-derived migrasomes promote cancer progression by fostering an immunosuppressive tumor microenvironment. Cancer Lett. 605, 217289. 10.1016/j.canlet.2024.217289 39389157

[B120] Zhu R. Zhou H. Chen W. Bai S. Liu F. Wang X. (2024). BCL2L1 is regulated by the lncRNA MIR4435-2HG-miR-513a-5p-BCL2L1 ceRNA axis and serves as a biomarker for pancreatic adenocarcinoma treatment and prognosis. Gene 925, 148615. 10.1016/j.gene.2024.148615 38788819

